# Oxygen Vacancy Formation on Boron-Contaminated Ti-V-Nb Non-Evaporable Getter Surfaces for Fusion Applications and Its Implications for Hydrogen Adsorption

**DOI:** 10.3390/nano16211392

**Published:** 2026-10-29

**Authors:** Alvin Kambondo, Jie Wang, Huang Pan

**Affiliations:** 1 Shaanxi Engineering Research Center of Advanced Nuclear Energy, Shaanxi Key Laboratory of Advanced Nuclear Energy and Technology, School of Nuclear Science and Technology, School of Energy and Power Engineering, Xi’an Jiaotong University, Xi’an 710049, China; 3123999189@stu.xjtu.edu.cn; 2 Southwestern Institute of Physics, Chengdu 610225, China; huangpan@swip.ac.cn

**Keywords:** non-evaporable getter, boron, hydrogen adsorption, fusion vacuum

## Abstract

The influence of boron surface contamination on oxygen activation and subsequent hydrogen adsorption on Ti-V-Nb non-evaporable getter (NEG) surfaces was investigated using density functional theory (DFT). A series of boron concentrations from 0 to 66.7 at.% was systematically introduced into the surface layer, spanning dilute perturbation to strong surface reconstruction regimes. Chemisorbed oxygen was fixed in a consistent configuration to simulate the passivating oxide overlayer formed under realistic conditions. Two independent performance criteria were considered. These were ease of activation, assessed by oxygen vacancy formation energy, and hydrogen pumping capability, assessed by H_2_ adsorption energy. The results reveal that moderate boron contamination at 41.7 at.% reduces the average oxygen vacancy formation energy by 1.232 eV, promoting easier oxygen removal, while excessive boron loading leads to oxygen trapping. Screening the concentrations by this descriptor therefore identifies 5B as the least detrimental to activation within the investigated range. On this surface, H_2_ adsorption is strongly site-dependent, with bridge and hollow sites exhibiting dissociative chemisorption. PDOS analysis shows that B 2*p* states mediate hydrogen activation either directly through B 2*p*-H 1*s* hybridization at the hollow site or indirectly by modifying the neighbouring metal centres at the bridge site, while the elevated work function of the 5B surface reflects enhanced surface dipole strength rather than a barrier to H_2_ adsorption. LST/QST calculations reveal a forward barrier of 0.422 eV for hydrogen migration from the surface into the subsurface, with the subsurface state thermodynamically more stable by 0.531 eV. These findings establish that moderate boron contamination does not severely hinder oxygen removal or eliminate hydrogen uptake, providing atomic-scale insight for fusion-relevant environments where boronization is routinely applied as a wall conditioning technique to suppress plasma contamination and sustain high-quality plasma confinement.

## 1. Introduction

Non-evaporable getter (NEG) technology based on transition metal alloys is an effective solution for ultra-high vacuum systems and has been adopted in magnetic confinement fusion devices, particle accelerators and other systems where efficient hydrogen pumping and impurity control are essential [1]. For fusion reactors, residual hydrogen and oxygen impurities released from vessel walls will pollute core plasma, degrade confinement performance and aggravate fuel loss, making maintaining long-term vacuum stability critical for reducing plasma contamination, minimising fuel loss and ensuring stable reactor operation [2,3]. Such impurities elevate the effective plasma charge [4]:

(1) $$Z_{\mathrm{eff}}=\sum_{i}\frac{n_{i}Z_{i}^{2}}{n_{e}},$$

where $n_{e}$ is the total electron density, $n_{i}$ is the density of ion species *i*, and $Z_{i}$ is the charge number of that species (e.g., +1 for deuterium, +8 for fully stripped oxygen). In a multi-species plasma, charge neutrality requires:

(2) $$n_{e}=\sum_{i}n_{i}Z_{i}.$$

which implies that at fixed total electron density, every impurity ion of charge *Z* replaces *Z* fuel ions, directly diluting the reacting deuterium and tritium. Additionally, the bremsstrahlung power radiated by a single impurity species is:

(3) $$P_{\mathrm{br}}=5.35\times 10^{-37}\;Z^{2}n_{e}n_{z}T_{e}^{1/2}\;W\;m^{-3},$$

with $T_{e}$ in keV. Summing over all ion species and using the definition of $Z_{\mathrm{eff}}$ yields the well-known scaling:

(4) $$P_{\mathrm{rad}}=5.35\times 10^{-37}\;n_{e}^{2}\;Z_{\mathrm{eff}}\;\sqrt{T_{e}}.$$

Thus, any ingress of high-*Z* impurities simultaneously reduces the fuel density and increases radiative energy loss, both of which degrade fusion performance. Mitigating impurity sources is therefore essential, and well-designed NEG thin films serve a primary vacuum-protection role to maintain stable plasma operation under long-pulse conditions. Among candidate materials, Ti-V-Nb-based alloys are particularly attractive due to their strong hydrogen affinity, chemical stability and compatibility with thin-film deposition technologies [5,6].

The operational performance of NEG coatings is governed by two intricately linked processes, rapid hydrogen adsorption and dissociation on the surface and sustained surface activation under realistic exposure conditions [7,8]. In practice, surface oxidation [9] constitutes one of the most severe limitations, as chemisorbed oxygen species substantially modify surface reactivity, occupy active sites and elevate the activation temperature necessary to restore hydrogen pumping capability [10]. Consequently, understanding and controlling hydrogen adsorption on these surfaces lies at the heart of advancing NEG coating technology [11].

From an atomistic perspective, oxygen removal during activation is closely linked to the stability of metal–oxygen bonds and the energetic cost of defect formation. The oxygen vacancy formation energy provides a fundamental descriptor of activation behaviour, quantifying the thermodynamic driving force required to liberate oxygen from the surface [12]. Lower vacancy formation energies correspond to weaker oxygen binding and thus more readily activatable surfaces, while higher values indicate stronger passivation and reduced pumping efficiency [12].

It is important to distinguish between the two performance metrics that govern NEG operation. Oxygen vacancy formation energy measures the thermodynamic cost of removing the passivating oxide layer, which controls the ease of activation. Hydrogen adsorption energy measures the strength of H_2_ binding and dissociation, which controls pumping capability. These two properties are not necessarily correlated, as previously noted for other catalytic systems where vacancy formation and adsorption were found to be only weakly coupled [13]. Therefore, this study treats activation and hydrogen uptake as separate performance criteria and evaluates them independently. In multi-component alloys such as Ti-V-Nb, hydrogen adsorption is highly site-dependent due to variations in local coordination environment and electronic structure [14,15,16]. Different surface terminations further introduce complexity, as each exhibits distinct adsorption geometries and charge redistribution patterns, resulting in a wide distribution of hydrogen adsorption strengths and distinct activation pathways [12,15].

In fusion environments, an additional and often overlooked factor is boron from boronization wall conditioning. Magnetic confinement fusion devices rely on ultra-clean plasma environments, and in-vessel NEG coatings together with boronization wall conditioning jointly determine plasma purity and long-duration operational stability, making the interaction between boron and getter surfaces a critical plasma-material interaction issue that urgently requires atomic-scale clarification. As reviewed by Winter [17], boronization reduces oxygen impurities through deposition of boron-rich coatings that trap oxygen via strong chemical affinity. While boron effectively getters oxygen, its incidental incorporation into near-surface regions of NEG coatings represents an environmentally driven surface contamination rather than an intentional alloying strategy. This raises the question of how boron-contaminated surfaces influence NEG oxygen gettering, since both boron and the NEG alloy compete for oxygen binding sites. Moreover, wall conditioning must simultaneously address plasma impurity control and hydrogen recycling [17]. Initial Large Helical Device (LHD) investigations show that while boronization does not impair NEG pumping performance, operational scenarios involving hydrogen and deuterium glow discharges remain to be refined [18]. Understanding how boron contamination affects hydrogen adsorption on NEG surfaces is therefore essential for assessing the compatibility of these two gettering systems in fusion devices.

Motivated by this realistic exposure scenario, we investigate how boron contamination affects oxygen activation on oxidized Ti-V-Nb surface and whether the most readily activatable surface remains functional for hydrogen adsorption. In practice, NEG coatings are never perfectly clean under operational conditions, rather, their surfaces are invariably passivated by chemisorbed oxygen, which blocks active sites and degrades hydrogen pumping performance. Activation is therefore required to remove this oxygen overlayer and restore gettering functionality [19]. Boron contamination from boronization may alter the stability of this surface oxide, either facilitating or hindering the oxygen removal process. To assess the influence of boron on activation behaviour, we fix chemisorbed oxygen configurations across all models and compute the oxygen vacancy formation energy as a primary descriptor. This allows us to evaluate how surfaces with varying boron concentrations respond to oxygen removal. The configuration exhibiting the lowest vacancy formation energy is identified as the most readily activatable surface under boron-exposed conditions.

For this representative system, we further investigate hydrogen adsorption energetics, electronic structure modification and charge redistribution to verify that the activation benefit is not accompanied by a loss of hydrogen pumping capability. This analysis does not aim to establish an independent hydrogen-adsorption optimum across boron concentrations; rather, it tests whether the activatable surface remains functional for hydrogen uptake. To provide a mechanistic description of hydrogen transport, LST/QST calculations are performed to evaluate hydrogen diffusion pathways and associated kinetic barriers within the chemisorbed surface.

## 2. Computational Methodology

### 2.1. Density Functional Theory (DFT) Calculation Parameters

DFT calculations were carried out using a plane-wave basis set with a 450 eV kinetic energy cutoff, a 1×1×1 k-point mesh and on-the-fly ultrasoft pseudopotentials for electron–ion interactions, while exchange-correlation effects were described by the Perdew–Burke–Ernzerhof (PBE) GGA functional [20,21,22,23]. This cutoff follows previous DFT studies on transition-metal nitride systems, where it was found sufficient to obtain well-converged structural and energetic properties [24]. Convergence tests for the present Ti-V-Nb-B-O system confirmed that this value is sufficient for the properties of interest, and it was used consistently for all structural optimizations and adsorption calculations. All calculations were non-spin-polarized, consistent with the paramagnetic behaviour reported for Ti-based and Ti-Nb alloys [25]. To capture the structural disorder inherent to the amorphous Ti-V-Nb getter alloy, an amorphous modelling strategy was adopted. A large set of candidate structures possessing the target composition and density was generated, and the configuration with the lowest energy was identified through Monte Carlo screening of over one thousand candidates within the Amorphous Cell framework.

The optimized amorphous cell contains 57 atoms in total, arranged in four layers with a composition of 15 Ti, 16 V, 18 Nb, and 8 O. This base cell was then used to create five models with different boron concentrations, as described in Section 2.2. The simulation cell is periodic in the y and z directions, and a vacuum thickness of 10 Å was introduced along the x direction to prevent artificial interactions between periodic replicas. The surface normal is defined along the x-axis of the amorphous simulation cell. This direction is labelled [100] for convenience, but it does not correspond to a crystallographic Miller index, since an amorphous solid lacks long-range crystallographic order. The top surface layers were fully relaxed, while the bottom layers were constrained to their bulk positions to preserve bulk-like behaviour.

Structural relaxations were carried out using the Broyden–Fletcher–Goldfarb–Shanno (BFGS) algorithm [26]. Convergence was deemed sufficient when the total energy change fell below $1\times 10^{-6}$ eV/atom and the residual forces on all atoms dropped below the specified force threshold, guaranteeing fully relaxed geometries prior to energetic analysis. The computational protocol was validated by optimizing an isolated H_2_ molecule, which gave a bond length of 0.741 Å, in close agreement with the experimental value of 0.74 Å [27].

### 2.2. Adsorption and Vacancy Formation Energies

**Figure 1:**
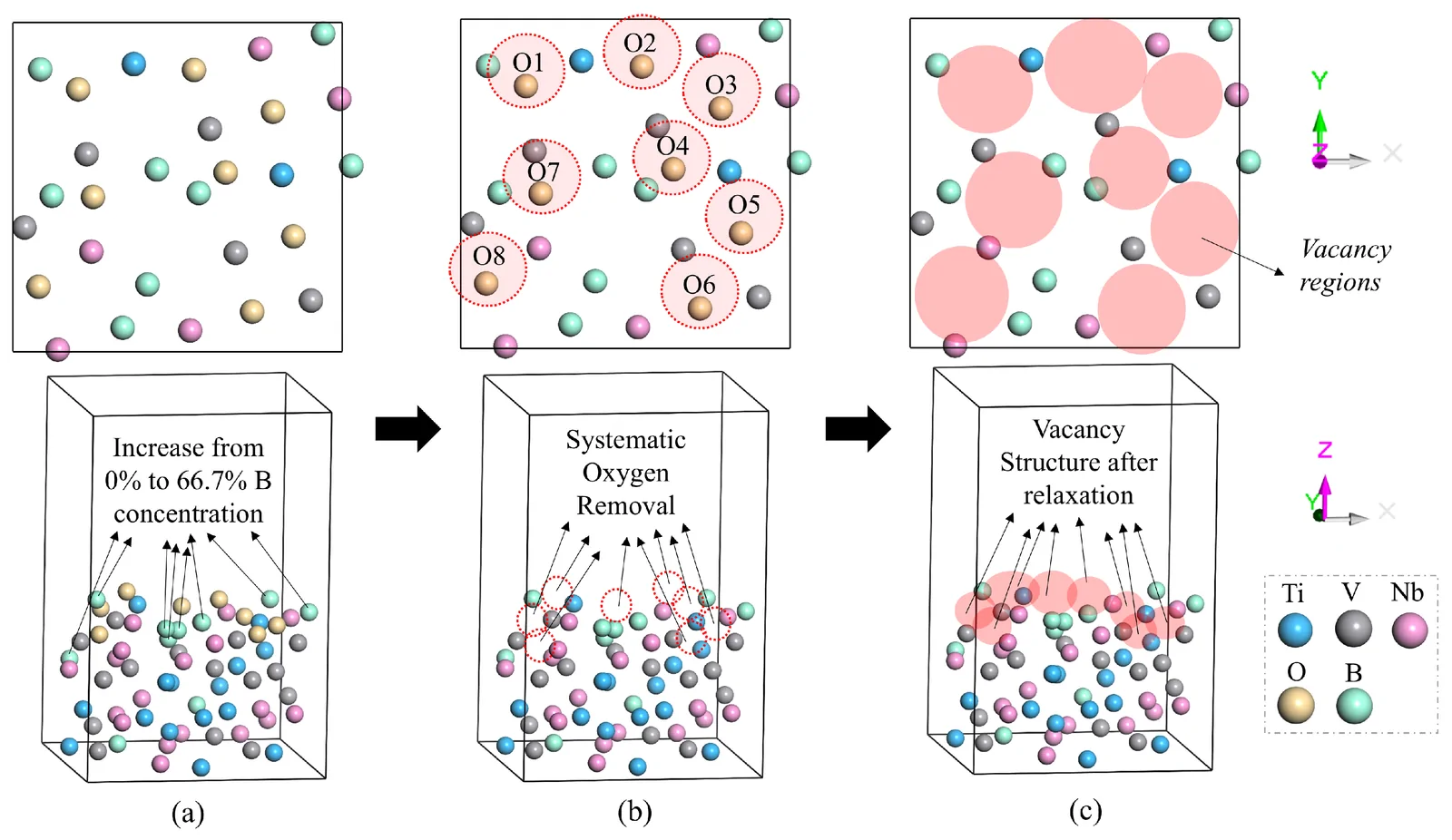
(**a**) Boron concentration series from 0 to 66.7 at.%, (**b**) systematic oxygen removal from oxidized surfaces for vacancy formation calculations, and (**c**) representative relaxed oxygen vacancy structure.

Surface boron alloying was introduced by systematically adding boron atoms onto the topmost atomic layer of the Ti-V-Nb-O surface as shown in Figure 1a, with the selected boron concentrations of 0.0, 8.3, 25.0, 41.7 and 66.7 at.% chosen to systematically span the full range from the clean surface through dilute perturbation, moderate alloying, strong alloying and very strong surface reconstruction regimes, as summarized in Table 1. The oxidized surface used for all vacancy calculations contains 8 chemisorbed oxygen atoms on the topmost layer, corresponding to a coverage of 8 O atoms per surface unit cell. These oxygen atoms were kept fixed in a consistent configuration across all boron concentrations to isolate the effect of boron on oxygen removal. The oxygen/metal coordination environments are described in detail in Section 3.1.

The boron concentration on the topmost surface was defined as:

(5) $$c_{B}=\frac{N_{B}}{N_{\mathrm{surf}}}\times 100\%,$$

where $N_{B}$ is the number of added boron atoms and $N_{\mathrm{surf}}=12$ is the number of metal atoms in the topmost surface layer of the amorphous slab. Thus, 5B means 5 boron atoms added among these 12 surface metal atoms, yielding a concentration of 41.7 at.%, and 8B means 8 boron atoms among the 12 surface metal atoms, yielding 66.7 at.%. All configurations were generated under identical addition protocols to ensure that observed trends originate from boron concentration effects rather than geometric bias.

Two independent performance metrics were evaluated in this study. The first is the ease of activation, quantified by the oxygen vacancy formation energy. The second is the hydrogen pumping capability, quantified by the H_2_ adsorption energy on the selected surface. These two metrics are not necessarily correlated, and they are therefore treated separately.

Oxygen vacancy formation energies were computed for oxidized surfaces according to [28]:

(6) $$E_{\mathrm{vac}}=E_{\mathrm{slab}\text{−}\mathrm{vac}}+\frac{1}{2}E_{O_{2}}-E_{\mathrm{slab}},$$

where $E_{\mathrm{slab}\text{−}\mathrm{vac}}$ is the energy of the slab containing an oxygen vacancy, $E_{O_{2}}$ is the energy of an isolated oxygen molecule, and $E_{\mathrm{slab}}$ is the energy of the fully oxidized slab.

Lower vacancy formation energies indicate weaker oxygen binding and therefore easier oxygen removal during thermal activation. Since oxygen desorption is a key step in NEG reactivation, the vacancy formation energy was used to identify the boron concentration that most readily facilitates activation. The configuration exhibiting the lowest average vacancy formation energy was selected for subsequent hydrogen adsorption and electronic structure analysis. This selection does not assume that the surface with the most favorable activation behavior also exhibits superior hydrogen adsorption performance. Rather, it provides the most readily activatable surface on which to test whether hydrogen pumping capability is preserved.

Hydrogen adsorption energies on the selected surface were calculated as [29]:

(7) $$E_{\mathrm{ads}}=E_{\mathrm{surface}\text{+}H_{2}}-E_{\mathrm{surface}}-E_{H_{2}},$$

where $E_{\mathrm{surface}\text{+}H_{2}}$ is the total energy of the adsorbate-surface system, $E_{\mathrm{surface}}$ is the energy of the relaxed clean surface, and $E_{H_{2}}$ is the energy of the isolated hydrogen molecule. A more negative $E_{\mathrm{ads}}$ indicates stronger binding and therefore more effective hydrogen pumping.

### 2.3. Electronic Structure Analysis

Electronic properties were characterised using electron density difference (EDD) maps, partial density of states (PDOS), work function calculations and d-band centre analysis. Charge redistribution associated with boron contamination and hydrogen adsorption was visualised using EDD maps calculated from the corresponding relaxed atomic configurations. Quantitative charge transfer and orbital overlap were further evaluated using Mulliken population analysis, including atomic charges and Mulliken overlap populations where applicable. All single-point electronic analyses were performed using the corresponding relaxed geometries to ensure that the calculated electronic properties reflected the optimized atomic configurations. For hydrogen adsorption, these analyses were used to examine changes in charge redistribution, orbital hybridization and chemical interactions following H_2_ adsorption and dissociation.

The influence of boron contamination on the electronic structure relevant to surface activation was evaluated using work function shifts, charge redistribution and the transition-metal d-band centre. These descriptors were used to examine how boron modifies the electronic environment of the oxygen-containing Ti-V-Nb surface and its relationship with oxygen removal during thermal activation. In contrast, the electronic origin of hydrogen adsorption and dissociation was examined using PDOS, EDD maps and Mulliken population analysis, while the corresponding hydrogen penetration pathways were further evaluated through LST/QST transition-state calculations, as described in the subsequent section.

## 3. Results and Discussion

### 3.1. Oxygen Vacancy Formation and Boron Concentration Effects

To quantify the influence of boron contamination on activation efficiency, oxygen vacancy formation energies were calculated for the oxidized Ti-V-Nb surface at different boron concentrations. In this study, boron was incorporated exclusively into the outermost atomic layer, and the boron concentration was defined relative to the number of metal atoms in that surface layer. This configuration represents a boron-rich layer deposited on the vacuum-facing surface of the NEG coating, while the underlying alloy remains largely unaffected. The calculations therefore focus on the surface chemistry governing oxygen removal during activation and subsequent hydrogen uptake, rather than on bulk alloying effects.

Lower oxygen vacancy formation energies indicate weaker oxygen binding and therefore facilitate oxygen removal during thermal activation. Table 2 summarizes the calculated vacancy formation energies for all oxygen sites. For the pristine surface without boron, the vacancy formation energies range from 4.597 to 5.802 eV, with an average value of 5.193 eV, indicating relatively strong oxygen binding across the surface. Boron contamination produces a concentration-dependent and non-monotonic modification of oxygen binding. At low boron concentrations, the changes in the average vacancy formation energy are relatively small. The average decreases from 5.193 eV for the boron-free surface to 4.982 eV at 1B and 4.997 eV at 3B, corresponding to reductions of 0.211 and 0.196 eV, respectively. The limited change in the average value indicates that a small amount of boron does not substantially weaken oxygen binding across the entire surface. Nevertheless, the site-specific values already reveal localized effects. At 3B, O7 and O8 exhibit the largest reductions of 0.892 and 0.610 eV, respectively, while O3 and O4 also show smaller reductions despite having no direct boron neighbors. Thus, the influence of boron is not restricted to oxygen atoms directly coordinated to B.

A substantially stronger modification occurs at 5B (41.7 at.% B). The average vacancy formation energy decreases to 3.960 eV, corresponding to a reduction of 1.232 eV relative to the boron-free surface. The minimum vacancy formation energy also decreases from 4.597 to 3.015 eV. These changes indicate that the 5B configuration facilitates oxygen removal considerably more effectively than the lower-boron configurations investigated. This behaviour is consistent with previous reports that boron contamination can substantially lower the oxygen vacancy formation energy. For example, in boron-doped defective ZrO_2_, introducing a B atom greatly reduced the vacancy formation energy from 5.52 to 4.85 eV [30]. The effect is also strongly site dependent. O2, O4, O5, O7, and O8 exhibit reductions of 2.001, 1.736, 1.658, 1.579, and 2.204 eV, respectively, with O8 showing the largest decrease.

Increasing the boron concentration to 8B does not provide any further reduction in oxygen binding. The average vacancy formation energy rises to 4.908 eV, which corresponds to a net reduction of only 0.285 eV relative to the boron-free surface, and the minimum vacancy formation energy increases from 3.015 eV at 5B to 4.348 eV at 8B. Within the limited set of configurations considered here, the 5B configuration gives the lowest average vacancy formation energy of 3.960 eV, but it also exhibits the largest site-to-site scatter (SD = 1.13 eV, compared with 0.40 eV at 0B and 0.38 eV at 8B). Consequently, the 5B average is less well constrained than those of the other compositions. Because only one boron placement was sampled per concentration and only the outermost layer was allowed to relax, the amorphous network cannot fully reorganize around the incorporated boron. These trends should therefore be regarded as preliminary and may partly reflect differences in local structural rearrangement rather than boron content alone.

The concentration dependence suggests two competing effects. Moderate boron contamination weakens selected metal–oxygen interactions through electronic modification, while higher boron loading stabilizes some oxygen atoms through local B-O coordination, and the balance between these effects determines the vacancy formation energy. To examine the origin of the site-to-site variation, the local coordination environment of each oxygen atom was analyzed further. Table 3 summarizes the coordination environment, number of B neighbors, and number of Nb neighbors for each oxygen site at 3B, 5B and 8B.

At 3B, O7 and O8 are the only oxygen sites with direct B neighbors and exhibit the largest reductions in vacancy formation energy. O7 has two B neighbors and its vacancy formation energy decreases by 0.892 eV, while O8 has one B neighbor and decreases by 0.610 eV. However, O3 and O4 also exhibit smaller reductions despite having no direct B neighbors. This indicates that the effect of boron extends beyond the oxygen atoms directly coordinated to B. The behavior at 5B provides stronger evidence for this nonlocal contribution. O2, O4, and O5 have no direct B neighbors in the 5B configuration, yet their vacancy formation energies decrease substantially relative to the 3B configuration. O4, which remains coordinated only by Ti and V, decreases from 4.424 to 3.015 eV, while O2 decreases from 5.209 to 3.155 eV despite having only one Nb neighbor.

These changes indicate that boron influences oxygen binding beyond its immediate coordination shell. In particular, the downward shift of the transition-metal d-band centre for the boron-contaminated surface, discussed in later sections, can alter the strength of metal–oxygen interactions, allowing oxygen binding to weaken even at sites without direct B coordination. The relatively small changes at O1, O3, and O6, which retain substantial Nb coordination, further suggest that the local metal composition also influences the response. Sites with greater Nb coordination remain comparatively resistant to oxygen removal, whereas Ti/V-rich environments show larger reductions in vacancy formation energy. This is consistent with the intrinsically stronger Nb-O bond. The Nb-O bond energy is as high as 753 kJ mol^−1^, substantially higher than that of other transition-metal–oxygen bonds [31].

The 8B configuration demonstrates the competing effect of increased local boron coordination. O4, which has no B neighbors at 5B, acquires two B neighbors at 8B and its vacancy formation energy increases from 3.015 to 4.348 eV. This increase is consistent with stabilization of oxygen through stronger local B-O interactions. O7 changes from three B neighbors at 5B to two B neighbors and two Nb neighbors at 8B, accompanied by an increase in vacancy formation energy from 3.018 to 4.462 eV. O2 and O8 also become associated with more Nb-rich environments and show increases in vacancy formation energy to 5.461 and 4.805 eV, respectively. These site-specific changes demonstrate that increasing the total boron concentration does not necessarily increase the ease of oxygen removal because the local arrangement of B and the transition-metal species surrounding each oxygen atom becomes increasingly important.

The calculated results, however, should be interpreted in terms of changes in surface bonding and electronic structure rather than a structural phase change of boron during activation. Boron has a high melting point of approximately 2076 °C, whereas NEG activation is typically performed at substantially lower temperatures, commonly around 200–400 °C. Melting of boron is therefore not expected under the activation conditions considered here. The calculated reduction in oxygen vacancy formation energy is consequently attributed to the modification of surface chemical bonding and electronic structure produced by boron contamination.

Based on the oxygen vacancy analysis, the 5B configuration was selected for subsequent H_2_ adsorption calculations because it exhibits the lowest average vacancy formation energy among the investigated boron concentrations. It does not imply that 5B necessarily provides the strongest hydrogen adsorption or the highest hydrogen pumping capability. Rather, the subsequent H_2_ adsorption calculations determine whether the activation advantage associated with the 5B surface is compatible with the hydrogen uptake required for NEG operation.

**Figure 2:**
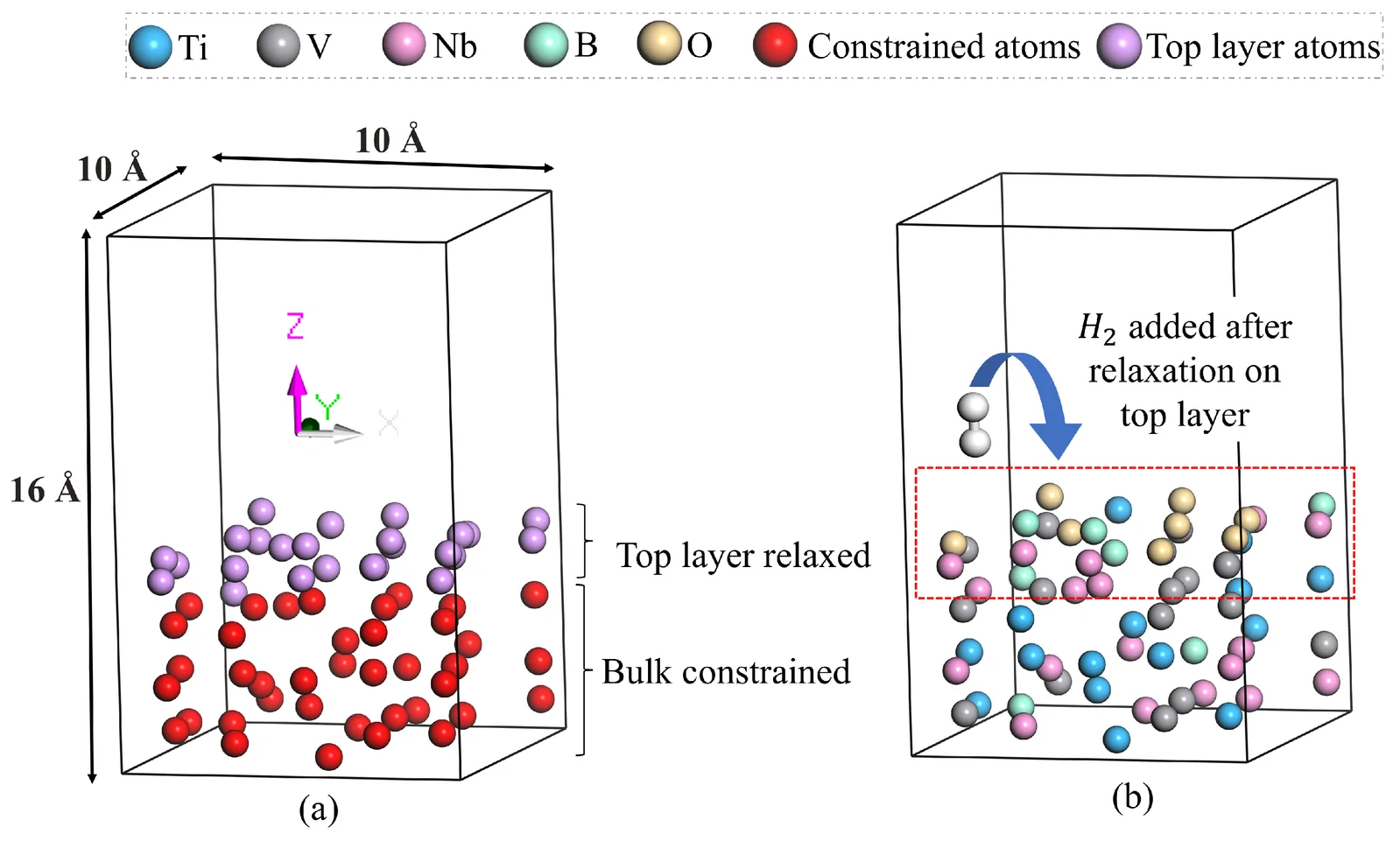
(**a**) Optimized slab structure showing relaxed surface layers and constrained bulk layers. (**b**) Boron-contaminated oxidized Ti-V-Nb surface with 41.7 at.% boron in the amorphous top layer selected for subsequent adsorption calculations.

The selected surface contains five B atoms in the amorphous top layer, corresponding to 41.7 at.% B within that surface layer only (see Section 2.2). Figure 2a shows the optimized slab geometry, with the upper surface layers fully relaxed and the lower layers constrained to preserve bulk-like behavior. Figure 2b shows the selected boron-contaminated surface containing five B atoms in the amorphous surface region, which was used for the subsequent adsorption calculations.

### 3.2. Hydrogen Adsorption on the 5B Surface Selected for Activation

For clarity in this work, ”hydrogen adsorption” refers to the interaction of H_2_ molecules with the surface, which may lead to dissociative chemisorption. Throughout this section, H_2_ denotes the gas-phase molecule, H denotes adsorbed atomic hydrogen, and H-H denotes the bond between the two hydrogen atoms. After identifying the boron concentration that has the least significant influence on activation (5B, 41.7 at.%), H_2_ adsorption calculations were performed on this surface to evaluate whether hydrogen adsorption capability is preserved after boron contamination. Strong hydrogen adsorption is essential for maintaining effective NEG pumping performance following activation.

**Figure 3:**
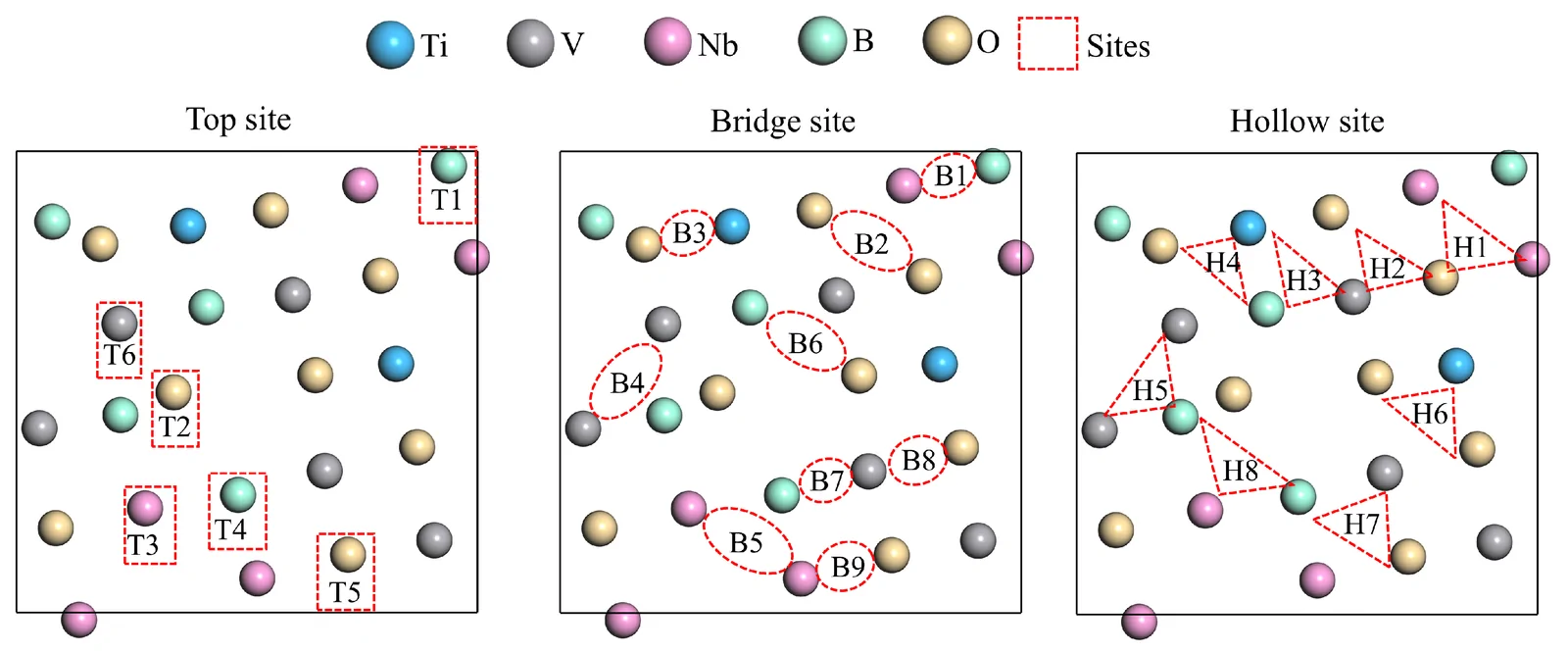
Adsorption configurations considered for H_2_ adsorption calculations, including top, bridge and hollow sites, showing only the top layer for clarity.

The adsorption configurations considered in this work are shown in Figure 3, including top, bridge, and hollow sites, which represent the major local coordination environments available on the oxidized boron-contaminated surface. It should be noted that although the hydrogen molecules were initially placed at idealized top, bridge and hollow positions before geometry optimization, the relaxed oxidized surface is not atomically flat due to the amorphous nature of the top layer. Consequently, even when H_2_ is initially placed on a nominal top site, it may still interact with neighboring atoms after structural relaxation. The final adsorption behavior therefore reflects the local multi-atom coordination environment rather than a perfectly ideal adsorption geometry.

The calculated adsorption energies and H-H bond lengths for all calculated adsorption sites are summarized in Table 4. The results show strong dependence on local atomic coordination, indicating that hydrogen adsorption is governed by the combined chemical effects of boron, transition metals and chemisorbed oxygen. The average adsorption energies are −0.124 eV for top sites, −2.152 eV for bridge sites, and −1.922 eV for hollow sites, with an overall average of −1.543 eV. Bridge sites therefore exhibit the strongest average adsorption, and the majority of bridge and hollow sites remain strongly binding, confirming that dissociative H_2_ chemisorption is prevalent across the boron-contaminated surface.

Top-site adsorption is generally less favorable than bridge and hollow adsorption, with adsorption energies ranging from −0.035 to −0.484 eV. The most stable top-site adsorption occurs at T3, with an adsorption energy of −0.484 eV and an H-H bond length of 0.839 Å. This slight elongation indicates modest activation, but the molecule remains molecularly adsorbed. This behavior indicates that top-site adsorption alone provides limited dissociation capability unless the local environment strongly promotes charge transfer.

Bridge sites exhibit the strongest adsorption among all configurations, with adsorption energies ranging from −1.393 to −2.793 eV. The most stable adsorption occurs at B6, with an adsorption energy of −2.793 eV and an H-H bond length of 3.554 Å, indicating complete dissociation of the hydrogen molecule [32]. Strong adsorption is also observed at B7 (−2.697 eV) and B9 (−2.648 eV). The strongest bridge configurations consistently involve boron coordinated with oxygen or transition-metal atoms, particularly boron-oxygen environments. This suggests that boron does not act as an isolated adsorption center but instead modifies the local electronic structure of neighboring atoms, especially the chemisorbed oxygen, thereby creating highly reactive interfacial adsorption sites that facilitate charge transfer into hydrogen antibonding orbitals.

Hollow-site adsorption also shows significant variation, with adsorption energies ranging from −0.972 to −2.657 eV. The most stable hollow configuration is H8, with an adsorption energy of −2.657 eV and an H-H bond length of 2.305 Å, indicating elongation of the molecular bond and complete dissociation. Strong adsorption is also observed at H1 (−2.595 eV), H3 (−2.543 eV), and H2 (−2.237 eV). Similar to the bridge-site trend, favorable hollow adsorption frequently occurs in environments where boron is coordinated with oxygen or transition-metal atoms. In contrast, weak adsorption is observed at H4, with an adsorption energy of only −0.972 eV, showing that boron contamination does not uniformly enhance hydrogen adsorption across all sites. The surrounding chemical coordination remains the dominant factor controlling adsorption strength.

The adsorption results indicate that the 5B surface supports strong dissociative hydrogen adsorption, primarily through boron-mediated interfacial active sites in oxygen-containing local environments. Boron weakens oxygen binding during activation while simultaneously transforming oxygen-containing regions into reactive adsorption centers for hydrogen. Based on the adsorption analysis summarized in Table 4, T3, B6, and H8 were selected as representative top, bridge and hollow adsorption sites for subsequent electronic structure and charge transfer analysis because they exhibit the strongest adsorption within their respective coordination classes.

### 3.3. Charge Transfer and Mulliken Analysis

The Mulliken analysis was used to quantify charge transfer between hydrogen and neighboring surface atoms and to evaluate bond populations after adsorption. Changes in atomic charge reveal the direction and magnitude of electron transfer, while bond population values provide insight into bond strength and covalent interaction [33]. In particular, analysis of the H-H bond population allows direct evaluation of molecular activation and dissociation. A reduction in H-H bond population relative to free molecular hydrogen indicates weakening of the covalent bond due to electron donation into antibonding orbitals. Simultaneously, increased bond population between hydrogen and surface atoms indicates the formation of new adsorption bonds.

Comparison of the charge distributions before and after adsorption therefore provides insight into how boron, transition metals and chemisorbed oxygen collectively influence adsorption strength on the 5B surface. Particular attention is given to boron-oxygen coordinated environments because the adsorption energy analysis showed these sites to be the strongest within their respective coordination classes.

The results summarized in Table 5 show clear differences in charge transfer and bond character depending on the local coordination environment on the 5B surface. In the T3 configuration, the Nb atom exhibits a small increase in net charge (0.33 before and 0.38 *e* after adsorption), indicating minor electron redistribution at the metal center. The hydrogen atoms acquire small negative charges (−0.12 and −0.14 *e*), while the H-H bond population remains high at 0.97 and the H-H distance is 0.839 Å. This combination of a preserved bond population and short bond length indicates that the hydrogen molecule remains largely intact, with only weak perturbation from the Nb site. This is consistent with the weak adsorption energy (−0.484 eV) and limited dissociation observed at this top site.

Despite their nearly equal adsorption energies (−2.793 eV at B6 and −2.697 eV at B7), these two boron-containing bridge configurations exhibit fundamentally different bonding behaviour. At B7, boron becomes more negative (−0.57 to −0.65 *e*) and forms strong B-H bonds (populations 1.11 and 0.55; distances 1.217 and 1.356 Å), while V becomes more positive (0.61 to 0.85 *e*). The H-H distance increases to 2.117 Å with a population of −0.07, confirming complete dissociation, and the H charges are −0.28 and −0.05 *e*. B7 is therefore a direct B-H bonding configuration (Table 5). At B6, by contrast, the initial placement was not a true bridge site, as H_2_ was positioned too close to the metal atoms and migrated away from boron during relaxation. Boron and oxygen charges remain essentially unchanged (−0.60 to −0.59 *e* and −0.64 to −0.63 *e*), and the H atoms instead bond to Ti, V, and Nb with populations of 0.42, 0.41, 0.28, 0.26, and 0.11. The H-H distance reaches 3.554 Å, again indicating full dissociation, with H charges of −0.25 and −0.28 *e*.

The negligible change in boron net charge does not imply that boron is uninvolved, since Mulliken populations do not incorporate orbital energy information and are also basis-set dependent and non-unique, and can yield unphysical values [34,35,36,37]. The B6 charge should therefore not be over-interpreted. As will be seen in the EDD and PDOS analyses, charge depletion around boron and accumulation around neighbouring metals indicate that boron activates the metal centres without forming direct B-H bonds, demonstrating an indirect mediation mechanism in contrast to the direct B-H bonding at B7.

For the H8 configuration, one boron atom (1B) exhibits increased electron density from −0.57 to −0.71 *e*, while the second boron (2B) becomes less negative from −0.32 to −0.17 *e*. The hydrogen atoms gain negative charges of −0.19 *e* (H1) and −0.10 *e* (H2). The table shows a strong 1B-H2 interaction with a bond population of 1.00 and a short bond length of 1.257 Å, while the 2B-H1 interaction is moderate (population 0.23, distance 1.713 Å). Niobium-hydrogen bonds are weak (Nb-H1: 0.06, Nb-H2: −0.15). The H-H separation is 2.305 Å with a bond population of −0.03, indicating that the hydrogen molecule is fully dissociated and the separated H atoms exhibit a weak antibonding interaction.

Across the representative sites, the Mulliken analysis reveals a clear distinction between boron-containing and Nb-top environments. At the H8 hollow site, 1B accumulates negative charge upon hydrogen adsorption while 2B becomes less negative, and the H-H population becomes negative, indicating complete dissociation. At the B6 bridge site, boron mediates activation indirectly through the neighbouring metal centres rather than through direct B-H bonding. In contrast, the T3 Nb top site shows only weak charge redistribution and little H-H activation, consistent with its weak adsorption energy. This confirms that boron-mediated charge transfer is the dominant mechanism driving H-H bond activation on the 5B surface, whereas Nb top sites are considerably less active.

### 3.4. Electron Density Difference Analysis

The electron density difference (EDD), Δρ, was employed to characterise the charge redistribution induced by hydrogen adsorption, such that:

(8) $$\Delta \rho =\rho_{\mathrm{surface}\text{+}H}-\rho_{\mathrm{surface}}-\rho_{H},$$

where $\rho_{\mathrm{surface}\text{+}H}$, $\rho_{\mathrm{surface}}$, and $\rho_{H}$ correspond to the charge densities of the adsorbate–substrate complex, the 5B boron-contaminated oxidized TiVNb(100) surface, and the isolated hydrogen species, respectively.

Upon exposure to the surface, $H_{2}$ undergoes dissociative chemisorption at the bridge and hollow sites, evidenced by pronounced H-H bond elongation to 3.554 Å at the B-O bridge site and 2.305 Å at the Nb-B-B hollow site. In contrast, the Nb top site exhibits an H-H bond length of 0.839 Å, indicating only modest activation and predominantly molecular adsorption. At these dissociative sites, the elongation arises from significant spatial charge transfer between the H atoms and adjacent surface atoms, with electrons shifted from the substrate to the hydrogen atoms and a concurrent reduction of the local electronic density on the surface metal states.

**Figure 4:**
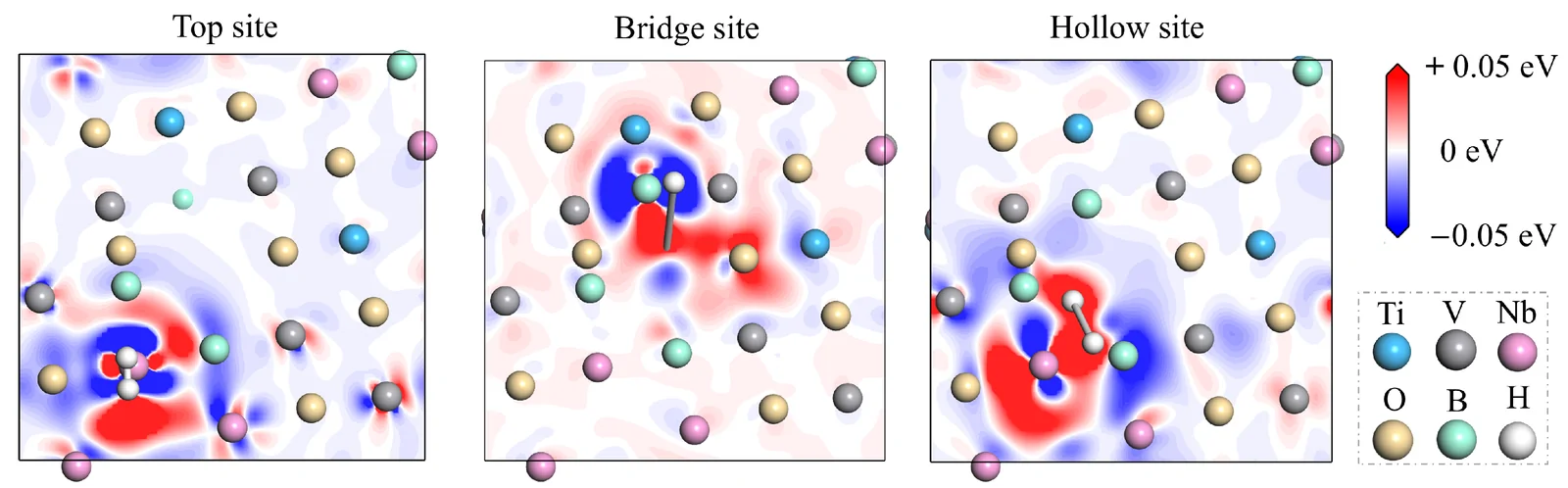
Two-dimensional electron density difference maps for the top, bridge and hollow sites, illustrating the charge transfer induced by hydrogen adsorption on the 5B boron-contaminated oxidized Ti-V-Nb surface. Red and blue areas represent charge loss and gain, respectively.

**Figure 5:**
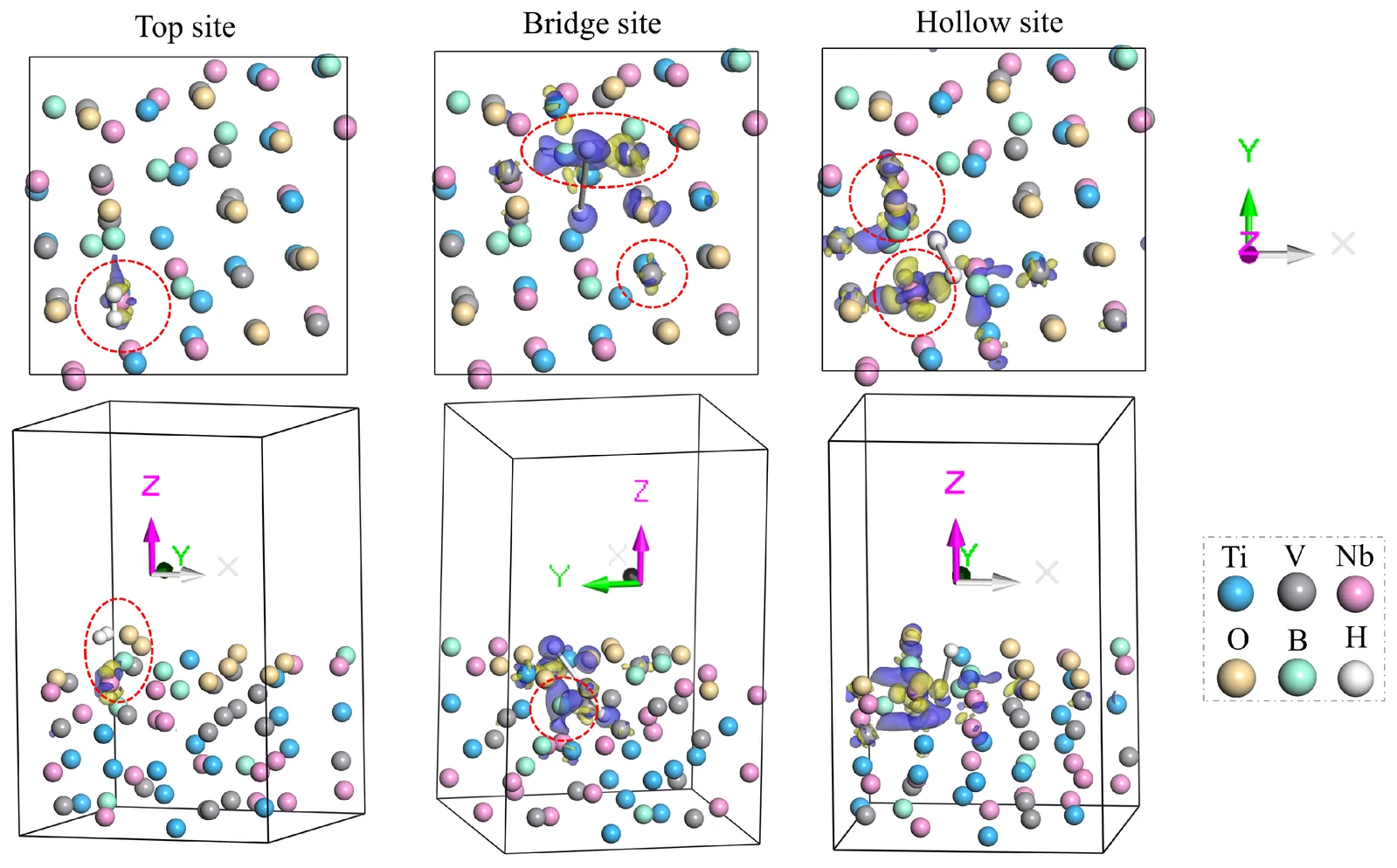
EDD for H_2_ adsorbed at the top, bridge, and hollow sites on the 5B surface. Yellow and blue isosurfaces represent charge accumulation and depletion, respectively (isovalue = 0.15 e Å^−3^). Red dashed circles indicate the hydrogen adsorption regions of interest.

To visualize this charge migration, two- and three-dimensional EDD maps are presented. Figure 4 displays the 2D contour profiles, where red and blue regions denote charge loss and gain, respectively. To further resolve the spatial orientation of this transfer, Figure 5 illustrates the 3D charge density difference at an isosurface value of 0.15 $e/{\text{Å}}^{3}$. Here, yellow regions correspond to charge accumulation, which is highly localized around the adsorbed H atoms, while blue regions indicate charge depletion zones situated predominantly on neighbouring metal atoms rather than on boron sites.

For the 5B configuration (41.7 at.%), EDD maps reveal enhanced charge transfer at the Nb-B-B hollow site, where the H-H bond reaches 2.305 Å, indicating complete dissociation. Boron acts as an electron acceptor, accumulating negative charge, while neighbouring Nb atoms become more positively charged, as confirmed by the Mulliken analysis. This redistribution creates electron-rich regions around the adsorbed hydrogen atoms and depletes the adjacent metal sites. At the bridge site, by contrast, boron mediates activation indirectly by modifying the neighbouring metal centres, as shown by the yellow accumulation around the metal atoms and blue depletion around boron. The 5B surface achieves a balance between two competing effects: higher boron concentrations (66.7 at.%) introduce excessive structural distortion and increase the oxygen vacancy formation energy, while the 5B concentration minimises it without severe surface strain or bonding disruption. The formation of stable surface-hydride complexes confirms that the 5B surface, selected for activation, remains functional for hydrogen gettering in fusion-relevant environments.

### 3.5. Partial Density of States Analysis

Partial density of states (PDOS) analysis provides a robust framework for evaluating orbital overlap and covalency between atoms. The overlap of partial states below the Fermi level can be used to assess the degree of orbital hybridization and the strength of covalent interactions [38]. The electronic structure of the surface prior to hydrogen adsorption is characterized by strong hybridization between O 2p states and transition-metal *d* orbitals of Ti, V and Nb across the −10 to −4 eV range. This interaction confirms the presence of a robust surface oxide layer and is responsible for the observed downshift of the *d*-band centre. Nevertheless, a significant density of metallic states persists in the vicinity of $E_{F}$, ensuring that hydrogen can still interact with specific local coordination environments.

**Figure 6:**
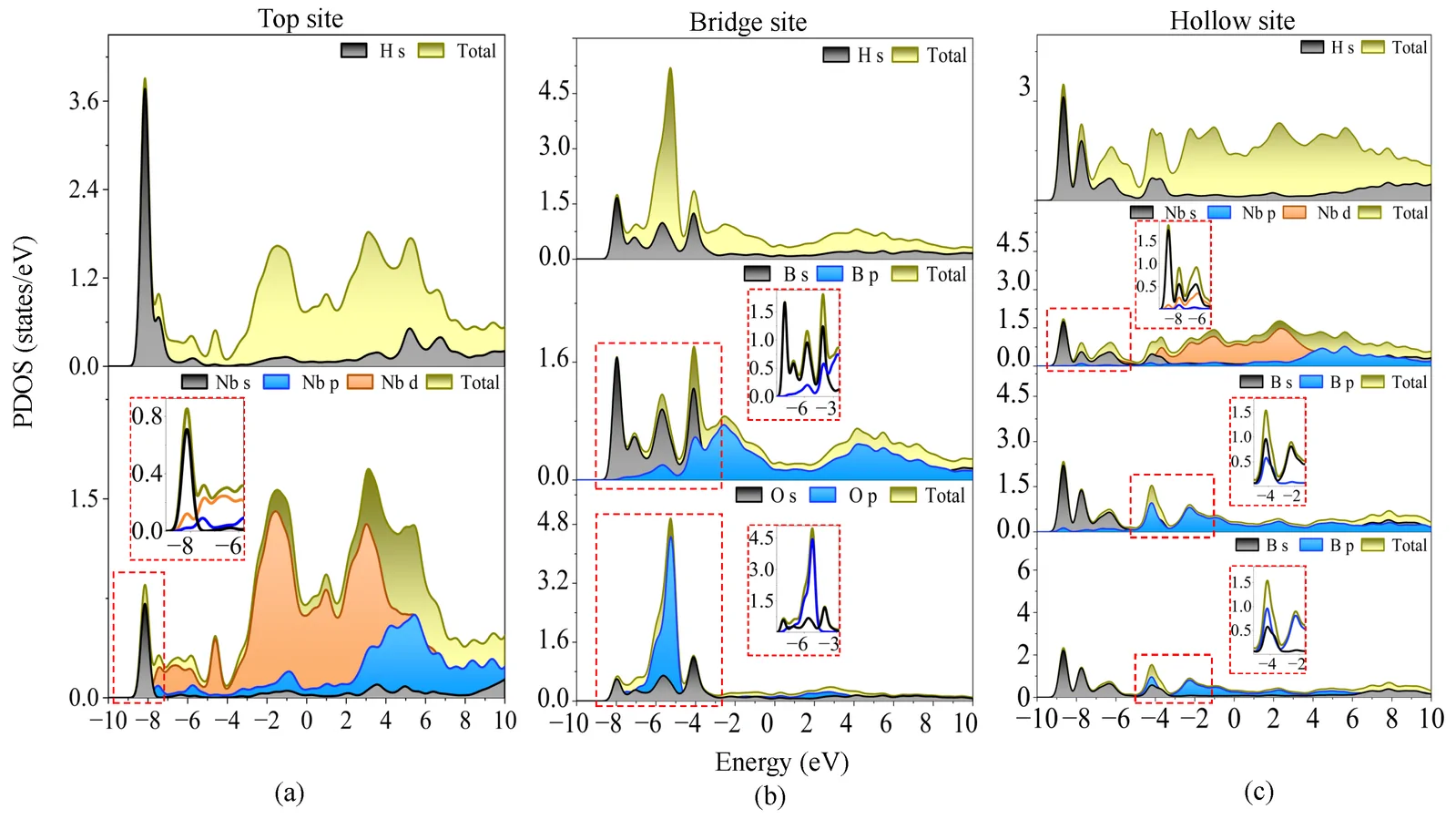
Partial density of states (PDOS) of (**a**) Nb top, (**b**) B-O bridge, and (**c**) Nb-B-B hollow sites on the 5B boron-contaminated oxidized Ti-V-Nb surface after H_2_ adsorption.

Hydrogen adsorption induces distinct electronic responses depending on the adsorption site. At the Nb top site (Figure 6a), the interaction is characterized by weak H 1s–metal *d* orbital mixing, with the H 1s states maintaining a sharp profile and showing only limited overlap with substrate states near $E_{F}$. This electronic configuration is consistent with molecular adsorption, where the H-H bond remains largely intact and surface-induced perturbation is minimal.

A fundamentally different behaviour emerges at the B-O bridge (Figure 6b) and Nb-B-B hollow (Figure 6c) sites, where extensive orbital hybridization drives H-H bond cleavage. For the bridge site, B 2p states lie further below the Fermi level, between −4 and −8 eV, and hybridize primarily with the metal *d* states in this region, while the H 1s states overlap with metal *d* states closer to the Fermi level. B 2p and H 1s do not share peaks at the same energy, indicating that boron influences hydrogen activation indirectly through the metal centres rather than through direct B-H orbital overlap. At the hollow site, B 2p states lie close to the Fermi level, between −1 and −4 eV, and share peaks with H 1s, while also hybridizing with the metal *d* states at deeper energies, affirming boron’s role as the direct orbital regulator at this site.

**Figure 7:**
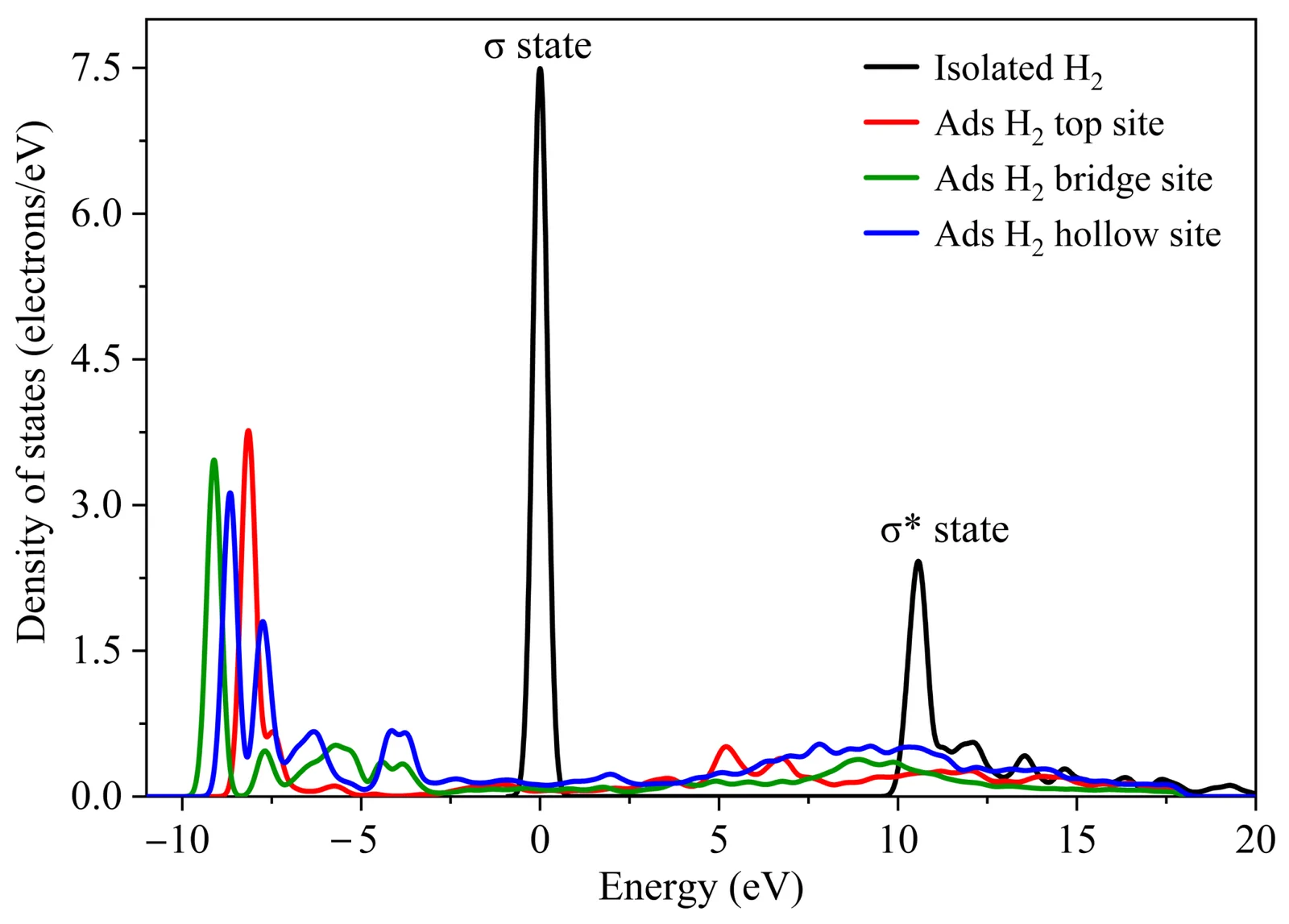
PDOS of isolated H_2_ and adsorbed H_2_ at different sites on the 5B boron-contaminated oxidized Ti-V-Nb surface. Broadening and shift of σ/$\sigma^{*}$ states reflect the intensity between H_2_ and surface atoms.

The evolution of the H_2_ molecular orbitals upon adsorption provides further insight into the activation mechanism. An isolated H_2_ molecule possesses a filled σ bonding state and an empty $\sigma^{*}$ antibonding state [39]. Following adsorption, the $\sigma^{*}$ state broadens and its intensity diminishes, signalling increasing interaction with surface states and the development of antibonding character. The σ state shifts downward in energy and undergoes slight broadening (Figure 7). These spectral modifications collectively indicate surface-induced perturbation of the H_2_ electronic structure and represent the onset of H-H bond activation.

A recurring observation at the hollow site is the dominance of B 2p states in the hybridization region. There, boron is directly bonded to the adsorbed hydrogen atoms, as confirmed by the Mulliken analysis, which shows substantial B-H bond populations. At the bridge site, B 2p states instead hybridize with the metal *d* states and the Mulliken analysis shows negligible B-H bond populations, indicating that boron regulates the electronic pathway indirectly through the neighbouring metal centres. The B 2p orbitals therefore govern the electronic pathway for hydrogen activation at both sites, explaining the substantially stronger adsorption observed at bridge and hollow sites relative to the top-site configuration. This boron-mediated electronic regulation, combined with the activation behaviour identified from the vacancy formation analysis, indicates that the 5B surface facilitates both oxygen removal and strong hydrogen uptake.

### 3.6. d-Band Centre and Electronic Descriptor Trends

The reactivity of the boron-contaminated oxidized Ti-V-Nb surface toward oxygen removal was rationalized within the framework of the d-band model. This approach was complemented by PDOS analysis to resolve site-specific orbital contributions. The d-band centre offers a macroscopic descriptor of metal–oxygen bond stability, while PDOS provides atomistic insight into the hybridization channels governing both oxygen removal and hydrogen activation. According to established d-band theory [40,41], a d-band centre positioned closer to the Fermi level enhances adsorbate–substrate coupling by increasing the availability of unoccupied states for orbital mixing. A downshift in the d-band centre correlates with diminished metal–oxygen hybridization and weaker oxygen binding.

The d-band centre was evaluated using the first moment of the partial d-state density:

(9) $$\varepsilon_{d}=\frac{\int_{-\infty }^{+\infty }E\;D_{d}\left(E\right)\;dE}{\int_{-\infty }^{+\infty }D_{d}\left(E\right)\;dE},$$

where $D_{d}\left(E\right)$ denotes the density of transition-metal *d* states of the top layer.

**Figure 8:**
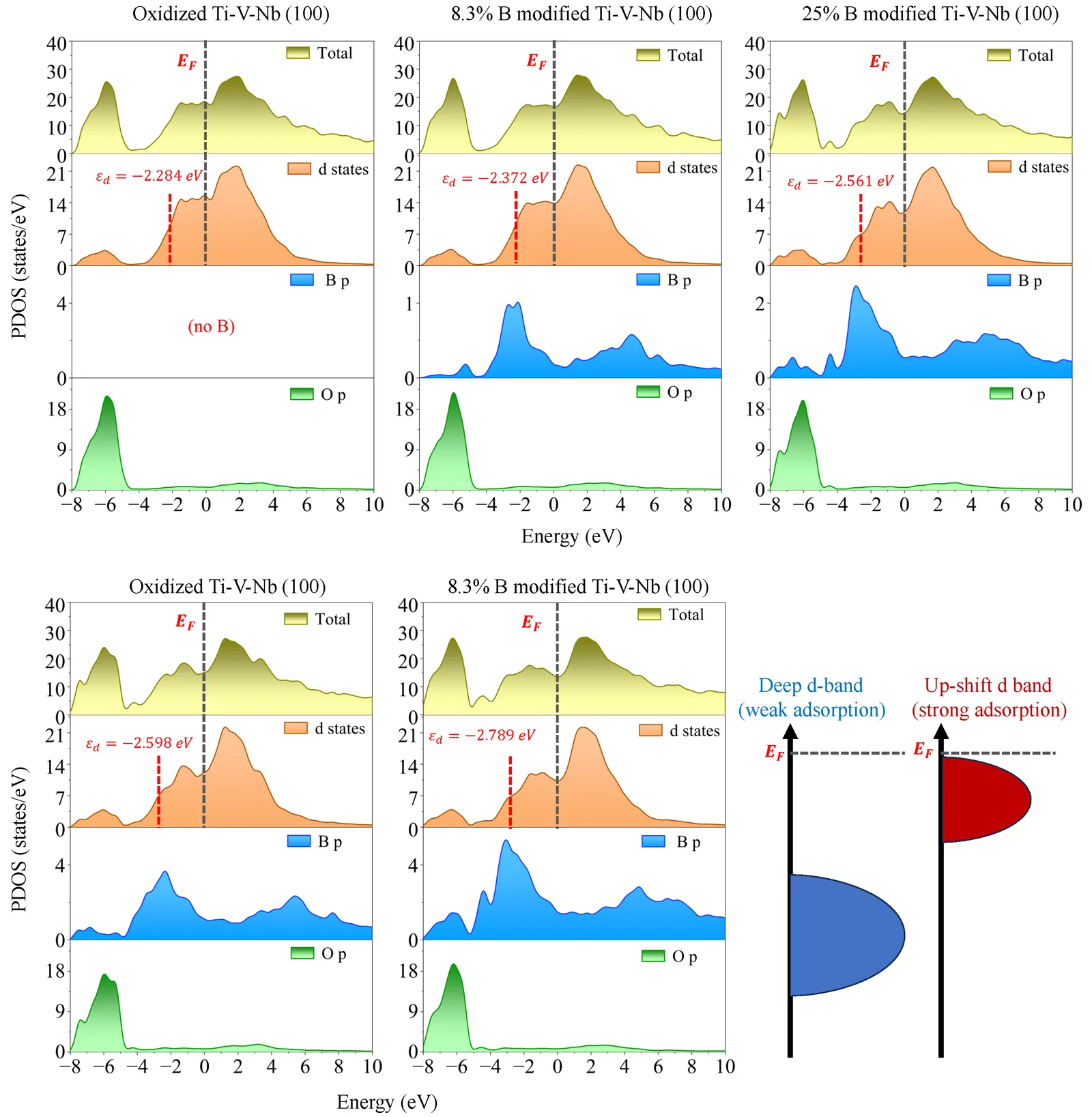
Evolution of the transition-metal d-band centre with boron concentration in the oxidized Ti-V-Nb surface.

The d-band centre of the transition-metal atoms in the top surface layer was calculated for each boron concentration. The pristine oxidized surface (0B) exhibits a d-band centre of (−2.284) eV. Boron contamination induces a progressive downward shift: (−2.372) eV at 1B, (−2.561) eV at 3B, (−2.598) eV at 5B and (−2.789) eV at 8B (Figure 8). The shift from 3B to 5B is relatively small, at only 0.037 eV, indicating that the electronic modification saturates at moderate boron loading. In contrast, the shift from 5B to 8B is more substantial at 0.191 eV, moving the d-band centre further away from the Fermi level. This indicates that the 8B surface has reduced availability of unoccupied metal d states for orbital hybridization.

The downward shift arises from enhanced *p*-*d* hybridization between B 2p and transition-metal *d* orbitals, which redistributes electronic density and lowers the d-state energy. Boron also hybridizes with oxygen through B 2p-O 2p interactions, further modifying the local electronic structure. This trend suggests progressively weaker metal–oxygen hybridization with increasing boron concentration, consistent with the reduced oxygen vacancy formation energies at moderate boron loading (Table 2). Lower d-band centres reduce electron donation into oxygen bonding states, weakening metal–oxygen bonds and facilitating oxygen removal.

However, oxygen vacancy formation does not follow a monotonic dependence on d-band position. Although the d-band centre decreases continuously from 0B to 8B, the average vacancy formation energy decreases only up to 5B (from 5.193 to 3.960 eV) before rising to 4.908 eV at 8B. This indicates that d-band position alone cannot fully explain oxygen removal behaviour. At low and moderate boron loading, boron acts primarily as an electronic modifier that weakens transition-metal–oxygen interactions. At higher loading, the surface undergoes significant structural changes due to boron-rich local environments (B-O, B-B-O and B-metal-O motifs), which can stabilize oxygen despite the continued downward shift of the d-band centre.

**Figure 9:**
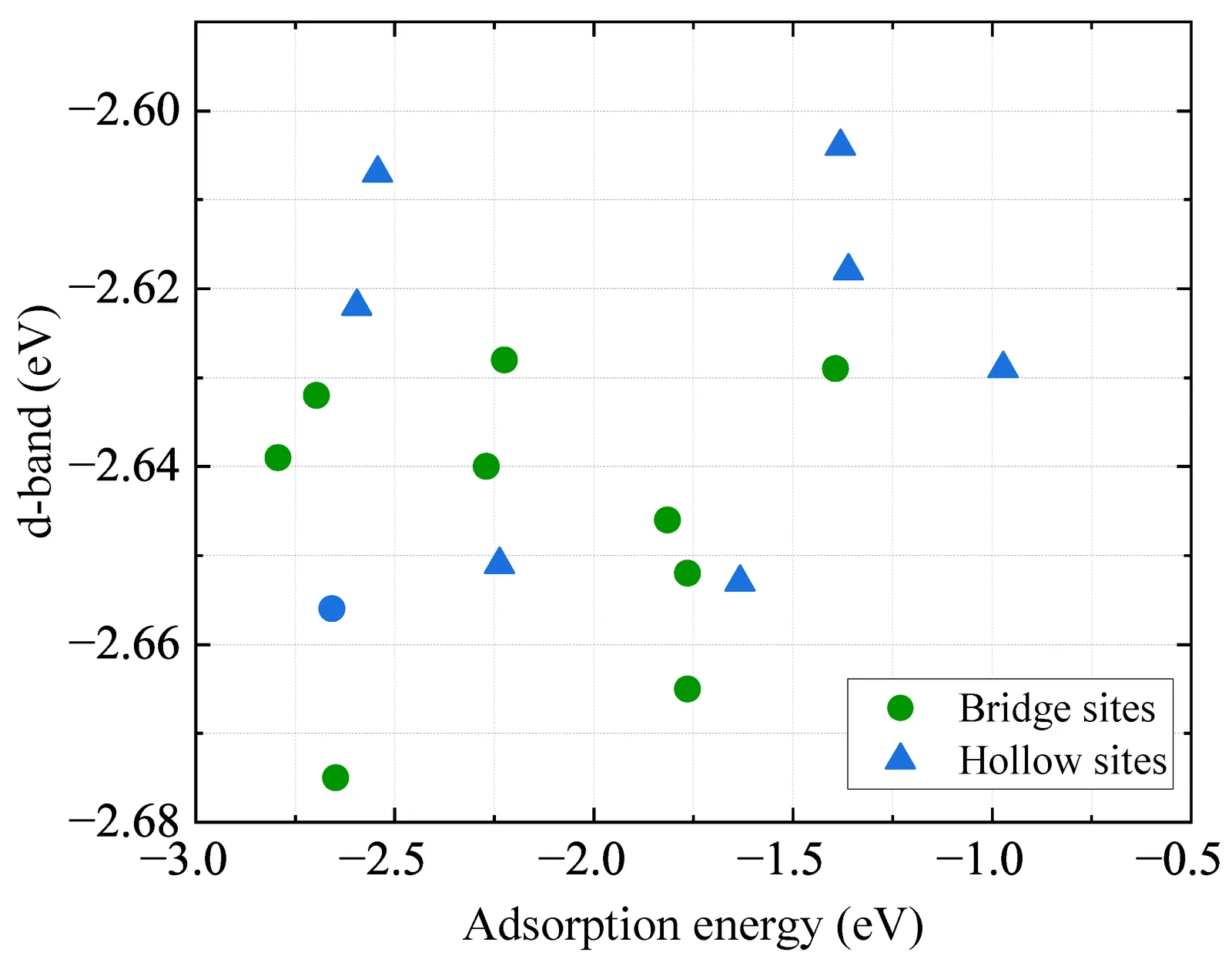
Site-projected d-band centre versus H_2_ adsorption energy for bridge and hollow adsorption configurations on the 5B boron-contaminated oxidized Ti-V-Nb surface indicating that hydrogen adsorption is governed by the local coordination environment rather than the average metal d-band position.

To resolve the site-specific electronic structure relevant to hydrogen adsorption, we computed the d-band centres for the atoms coordinating with hydrogen. Figure 9 plots the site-projected d-band centre against $E_{\mathrm{ads}}$ for the dissociative bridge and hollow sites, excluding the top site because H_2_ remains molecularly adsorbed. The site-projected d-band centres span a narrow range from −2.604 to −2.675 eV, while the adsorption energies vary widely from −0.972 to −2.793 eV. For the sampled sites, no clear correlation is observed between the site-projected d-band centre and the adsorption energy. For example, the strongest adsorption site B6 ($E_{\mathrm{ads}}=-2.793$ eV) has a d-band centre of −2.639 eV, whereas the weakest hollow site H4 ($E_{\mathrm{ads}}=-0.972$ eV) has a comparable d-band centre of −2.629 eV. Similarly, B7 ($E_{\mathrm{ads}}=-2.697$ eV) exhibits a d-band centre of −2.632 eV, which is closer to the Fermi level than that of B6, yet its adsorption is weaker.

These results demonstrate that the global d-band centre, which shifts monotonically with boron concentration, does not control hydrogen adsorption locally. Instead, the local B 2p states govern the electronic pathway at both sites, either through direct hybridization with H 1s at the hollow site or through hybridization with the metal *d* states at the bridge site, as shown by the PDOS analysis. The adsorption strength is therefore governed by the local coordination environment rather than the average metal d-band position. This decoupling explains why the d-band centre remains a useful descriptor for oxygen removal, which involves metal–oxygen bonds, but is not predictive for hydrogen activation, which is mediated by boron.

The 5B configuration represents a critical balance between these competing effects. Boron contamination is sufficient to weaken metal–oxygen hybridization while avoiding excessive boron-induced oxygen trapping, resulting in the lowest average vacancy formation energy and the least detrimental activation behaviour among the investigated configurations. In contrast, the 8B configuration increasingly resembles a boron-oxide transition-metal hybrid layer where oxygen stabilization through local boron coordination becomes dominant.

It is important to note that the d-band centre describes the transition-metal d states and therefore governs the metal–oxygen bonding that controls activation. It does not directly govern hydrogen adsorption on this surface. As shown by the PDOS analysis, H_2_ activation is mediated by B 2p states, either directly through B-H bonding at the hollow site or indirectly through boron-activated metal centres at the bridge site, rather than by the average metal d states. Consequently, the downward shift of the d-band centre with increasing boron concentration does not imply weaker H_2_ adsorption. The two processes are governed by different electronic states and are therefore decoupled. This distinction is consistent with the two-metric framework adopted in this study, in which activation and hydrogen uptake are evaluated independently.

These results demonstrate that the d-band centre remains a useful global descriptor for identifying trends in boron-induced modifications to metal–oxygen bonding. Its predictive capability becomes limited in highly oxidized amorphous multicomponent systems. In boron-contaminated Ti-V-Nb NEG films, oxygen removal is governed by the combined influence of transition-metal electronic structure, boron-induced charge redistribution and local atomic coordination, while hydrogen uptake is governed by the local coordination environment, through either direct B-H bonding or boron-activated metal centres.

### 3.7. Work Function Evolution with Boron Concentration and upon Hydrogen Adsorption

The work function provides insight into the electronic response of the oxidized Ti-V-Nb surface to both boron contamination and hydrogen adsorption, reflecting changes in the surface dipole induced by charge redistribution. Since the work function is directly related to the energy required to remove an electron from the surface into vacuum, its variation provides useful information regarding surface activation and adsorbate bonding. At T=0 K, the work function is formally defined as the minimum energy needed to extract an electron from the material [42], given by:

(10) $$\Phi =E_{\mathrm{vac}}-E_{F},$$

where $E_{\mathrm{vac}}$ is the electrostatic potential energy in the vacuum and $E_{F}$ is the Fermi energy of the slab. It should be noted that the work function is calculated for the entire slab, including the bulk region, and therefore inherits the systematic errors inherent to the DFT exchange-correlation functional. In addition, no dipole correction was applied in these calculations. Consequently, the absolute values should be interpreted with caution, while the relative trends between configurations, which are calculated on the same scale using Equation (10), remain meaningful.

**Figure 10:**
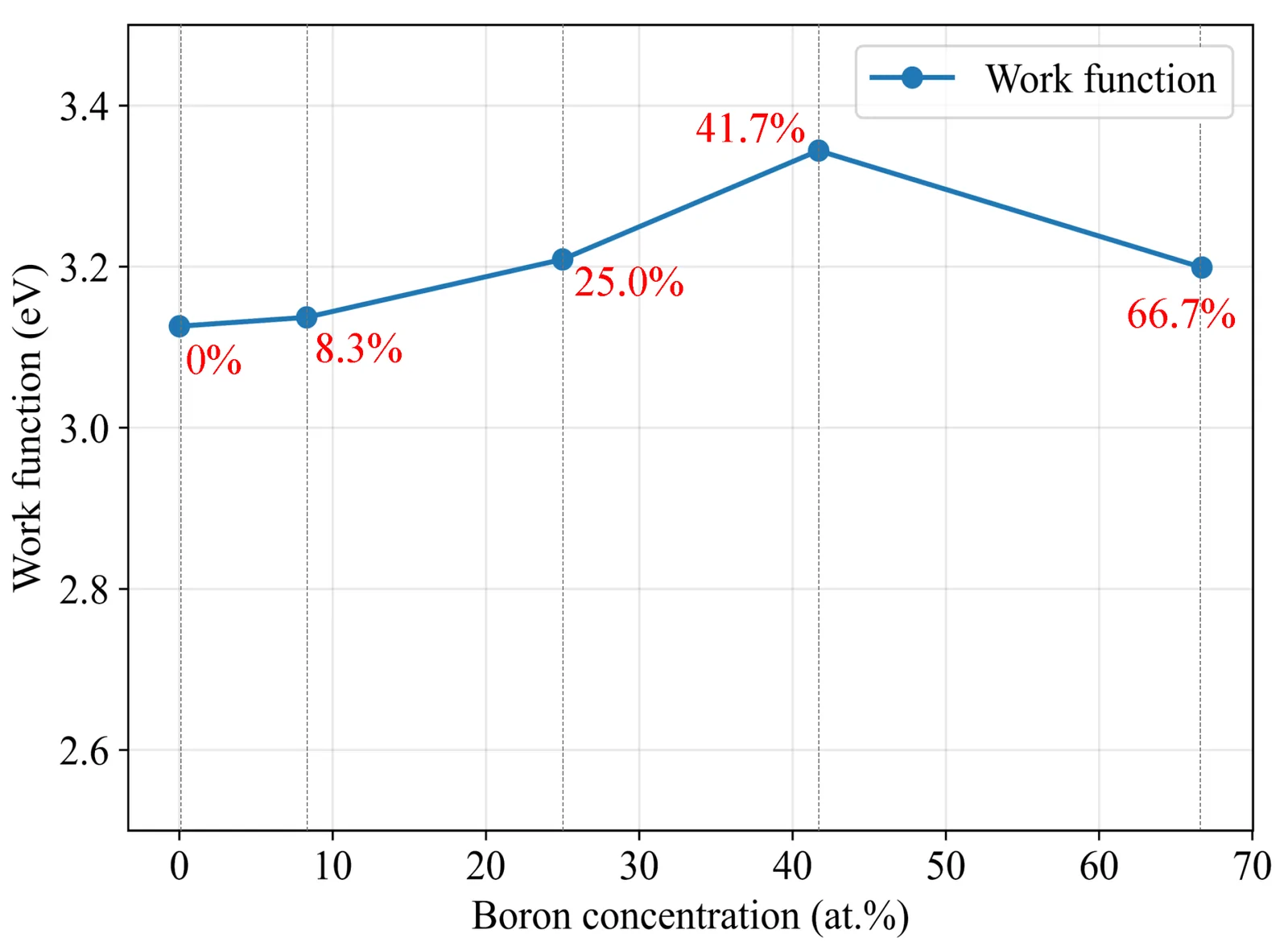
Evolution of work function as a function of boron concentration in the amorphous top layer of oxidized Ti-V-Nb. The work function increases with boron concentration up to 41.7 at.%, indicating enhanced surface dipole strength. At 66.7 at.%, the top-surface work function decreases to 3.199 eV but remains higher than the pristine value of 3.126 eV, indicating that boron continues to modify the surface dipole even at high loading.

Figure 10 shows the calculated work function as a function of boron concentration in the amorphous oxidized surface layer. The pristine oxidized Ti-V-Nb surface without boron exhibits a work function of 3.126 eV. Upon contamination with one boron atom, the work function increases slightly to 3.137 eV, indicating only minor perturbation of the surface electronic structure. Increasing boron concentration to three atoms produces a more noticeable rise to 3.209 eV, suggesting stronger charge redistribution within the oxidized layer. The highest work function is observed for the 5B configuration, reaching 3.344 eV. This concentration corresponds to the configuration containing 5 boron atoms within the amorphous top layer, equivalent to 41.7 at.% boron in that surface layer (see Table 1). The increase in work function indicates substantial modification of the surface dipole caused by boron-induced electronic redistribution.

Boron has a strong affinity for oxygen and alters the local bonding environment, resulting in enhanced polarization at the oxide-metal interface. A rising work function is typically associated with a more strongly oxidized surface and a higher energy barrier for oxide reduction, which would require a higher activation temperature to restore pumping capability. Assessed by this descriptor alone, 5B would therefore be the most detrimental to activation among the investigated concentrations. These two descriptors are not interchangeable. The work function reflects one-electron removal from the highest occupied state, whereas the vacancy formation energy reflects two-electron oxygen removal and also captures the metal–oxygen bond strength and structural reorganization upon reduction, making it the more complete measure of reducibility [43]. It is therefore the vacancy formation energy that governs the selection of 5B.

At higher boron loading, however, this trend changes. For the 8B configuration, the work function becomes strongly asymmetric, with values of 3.199 eV for the top surface and 2.911 eV for the bottom surface. This asymmetry indicates nonuniform charge redistribution and structural distortion caused by excessive boron accumulation. Instead of further strengthening the surface dipole, excessive boron over-saturates the surface, leading to uneven electronic polarization and possible localized charge trapping or dipole cancellation. These results show that boron concentration critically influences the electronic properties of oxidized Ti-V-Nb surfaces. Moderate boron contamination enhances the surface dipole and promotes oxygen destabilization, whereas excessive boron reduces electronic uniformity.

**Figure 11:**
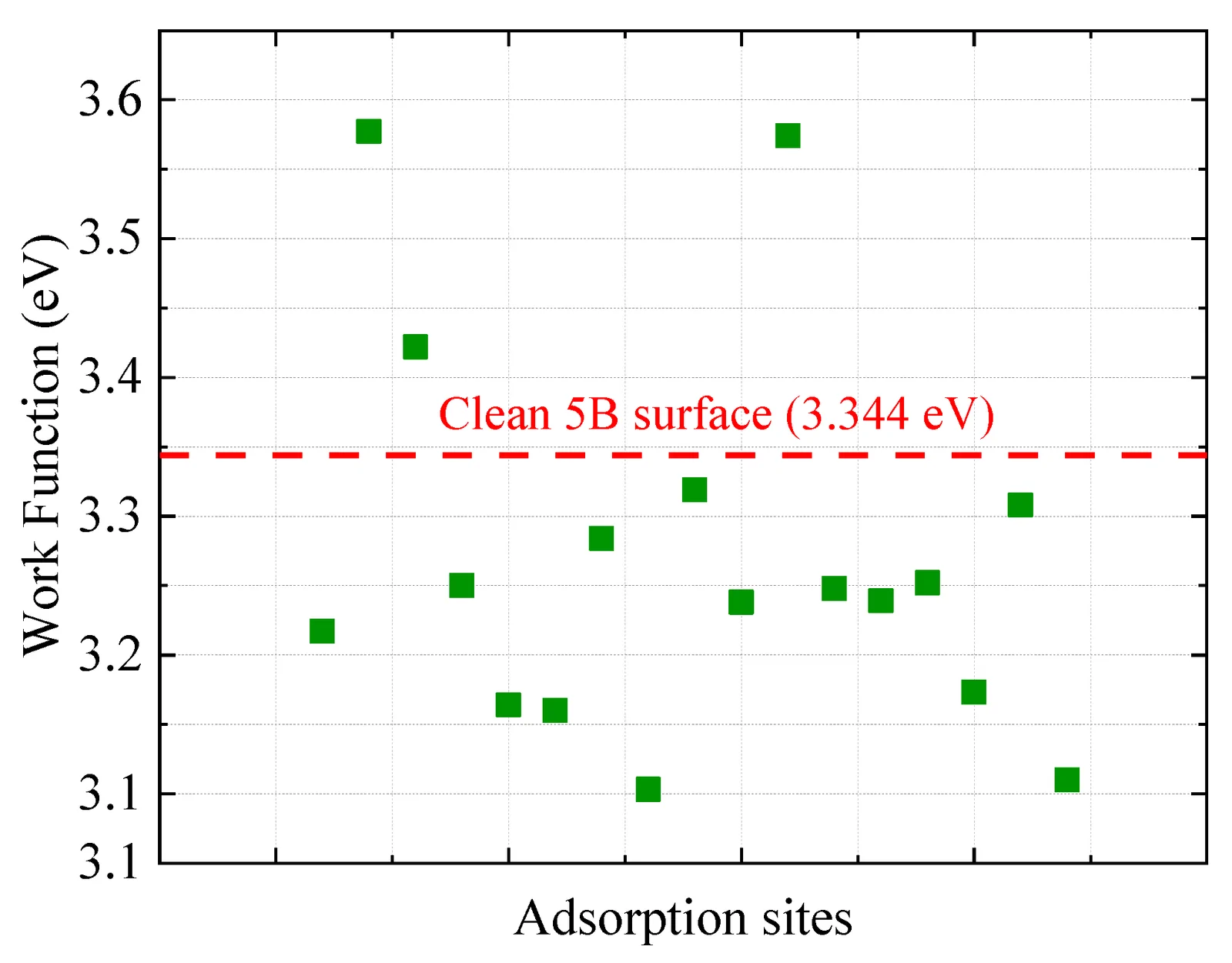
Top-surface work function for the bridge and hollow adsorption sites on the 5B boron-contaminated oxidized Ti-V-Nb surface. The red dashed line indicates the clean 5B surface work function (3.344 eV). Most sites lie below this line, showing that H_2_ adsorption generally decreases the work function, while a few sites show an increase due to local structural distortions.

To examine how H_2_ adsorption modifies the surface dipole, Figure 11 shows the top-surface work function on the 5B surface. The red dashed line marks the clean 5B work function value of 3.344 eV. Most sites lie below this line, indicating that H_2_ adsorption decreases the work function, while a few sites show an increase. The values range from 3.103 to 3.577 eV, with an average of 3.273 eV. This distribution reflects the heterogeneous local environments of the amorphous surface. The decrease arises from the disruption of the strong B-O surface dipoles that gave the clean 5B surface its high work function. When H_2_ dissociates, at the hollow site the strongly polar B-O bonds are partially replaced by less polar B-H bonds, while at the bridge site hydrogen bonds to the neighbouring metal atoms. Since H is less electronegative than O, the net surface dipole perpendicular to the surface is reduced, lowering the work function. This decrease does not indicate electron back-donation from hydrogen to the surface. The Mulliken analysis showed that hydrogen gains a small negative charge while the boron charge response is site-dependent, yet the overall dipole moment is reduced because the B-O contribution is diminished. The few sites showing an increase can be attributed to local structural distortions and inhomogeneous charge redistribution in the amorphous surface.

The relationship between work function and hydrogen adsorption in this system warrants further discussion. In the literature, a lower work function is often considered beneficial for H_2_ adsorption on metal surfaces, because it can facilitate electron donation from the surface into the H_2_ antibonding orbital. In the present system, the pristine surface exhibits the lowest work function (3.126 eV) among all top surfaces considered. As boron is incorporated, the work function increases progressively up to 5B (3.344 eV) and then decreases slightly at 8B (3.199 eV), though it remains above the pristine value at all boron concentrations. However, no H_2_ adsorption calculation was performed on the boron-free surface in this work. Therefore, a direct comparison of hydrogen adsorption strength between the pristine and boron-contaminated surfaces cannot be made from the present data. The work function trend alone does not establish that boron reduces the intrinsic hydrogen adsorption ability of the surface. It is therefore reported here as a descriptor of boron-induced surface dipole modification, not as a predictor of hydrogen adsorption strength.

The potential trade-off between these two performance metrics, ease of activation and hydrogen pumping capability, was therefore examined, and the present results show that the two effects are decoupled. Boron contamination facilitates oxygen removal while modifying the surface electronic structure. Among the investigated configurations, 5B provides the least detrimental balance of activation and hydrogen uptake, and its hydrogen adsorption capability remains strong enough to support dissociative chemisorption. The elevated work function of the 5B surface does not prevent H_2_ adsorption because the adsorption mechanism is mediated by local B 2*p* states, through either direct B-H bonding or boron-activated metal centres, rather than by global electron transfer from the Fermi level, as demonstrated by the PDOS analysis (Section 3.5). The two effects are decoupled because they involve different electronic states and different spatial regions of the surface.

The compatibility of boron contamination with NEG pumping performance must be assessed against experimental observations from fusion devices. In the Large Helical Device (LHD), in-vessel NEG pumps were operated under boronized conditions, and it was reported that “while the boronization and glow discharge under noble gases do not show effect on the pumping performances of the NEG pumps, the establishment of an operational scenario including hydrogen and deuterium glow discharges consistent with other pumping facilities is still an aspect to be refined” [18]. This experimental observation is consistent with the present calculations. On the 5B surface, dissociative H_2_ adsorption remains strongly favourable at bridge and hollow sites, with adsorption energies reaching −2.793 eV, indicating that boron contamination does not eliminate favourable hydrogen adsorption. The present calculations therefore support the LHD observation that boronization does not impair NEG pumping performance.

### 3.8. Transition Pathway Analysis

To further investigate hydrogen transport following surface adsorption, transition pathways between selected surface and subsurface configurations were calculated using the linear synchronous transit/quadratic synchronous transit (LST/QST) method [44]. While adsorption calculations identify thermodynamically favorable hydrogen adsorption configurations, they do not provide information about the kinetic barriers associated with subsequent hydrogen migration away from the surface. Because hydrogen uptake by non-evaporable getters involves transport beyond the initial surface adsorption state, evaluating the barrier for surface-to-subsurface migration provides additional information on whether strongly adsorbed hydrogen can enter subsurface sites. The LST/QST calculations were therefore performed to examine the migration behavior of hydrogen on the selected 5B boron-contaminated oxidized Ti-V-Nb surface.

The initial configuration consisted of two dissociated hydrogen atoms placed on the relaxed 5B boron-contaminated oxidized Ti-V-Nb surface. The reactant and product configurations were first fully optimized at the same level of theory and using the same slab model and exchange-correlation functional as those employed for the adsorption calculations. The product configurations were constructed by migrating the hydrogen atoms beneath the surface layer into selected subsurface positions. LST/QST calculations were then performed between the optimized surface and subsurface configurations to determine the corresponding transition states and activation barriers. These calculations provide a direct assessment of the kinetic accessibility of the selected hydrogen migration pathways, while the reaction energies indicate the relative thermodynamic stability of the resulting subsurface configurations.

**Figure 12:**
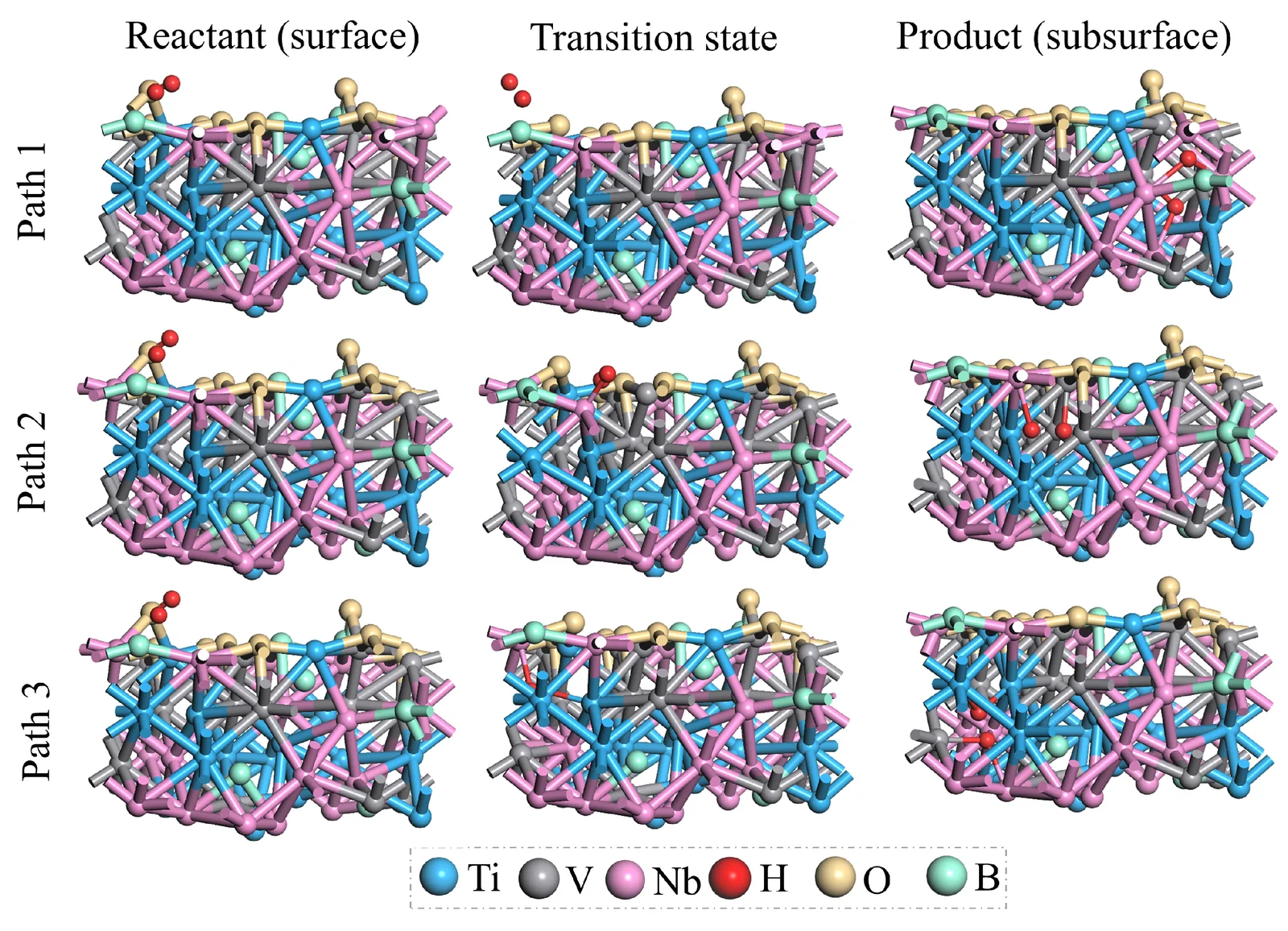
Geometries of the reactant, transition state, and product for the three hydrogen migration pathways on the 5B boron-contaminated oxygen-chemisorbed Ti-V-Nb surface.

**Figure 13:**
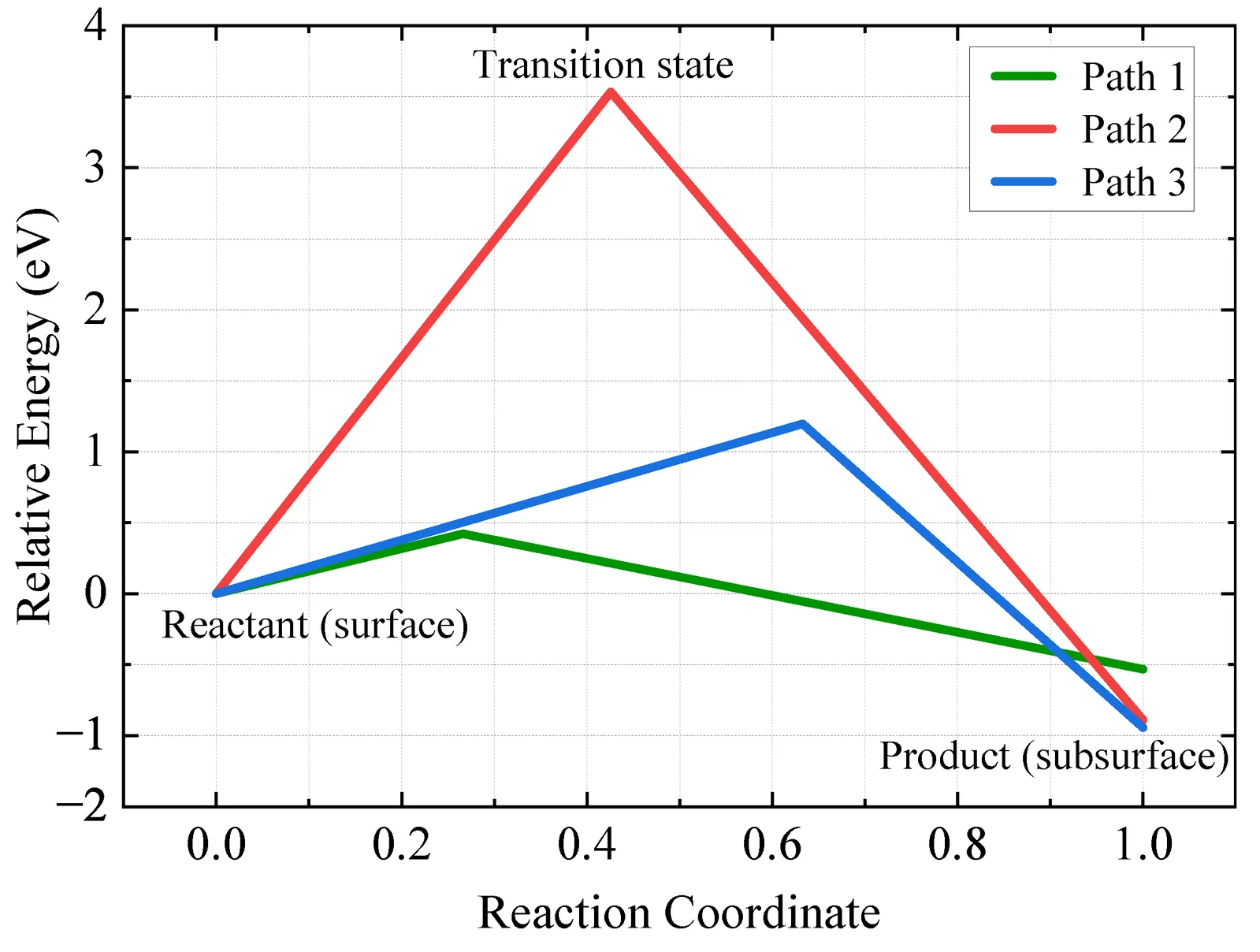
LST/QST energy profiles for hydrogen migration from the surface into the subsurface on the 5B boron-contaminated oxygen-chemisorbed Ti-V-Nb surface for Path 1, Path 2 and Path 3. The reactant energy is set to zero for each pathway.

Three distinct migration pathways were examined. Path 1 and Path 2 describe hydrogen migration within the first two surface layers, whereas Path 3 extends the migration into the fourth layer. The maximum number of QST steps was set to 7. The reactant and product structures were fully optimized using the same slab dimensions and computational parameters employed throughout the study. The RMS convergence criterion was set to 0.5 eV/Å, with a maximum of 20 conjugate-gradient iterations and 7 QST iterations. Figure 12 shows the optimized reactant, transition-state, and product configurations for the three calculated pathways. The corresponding LST/QST energy profiles are presented in Figure 13. The energy of the reactant configuration was set to zero for each pathway to facilitate comparison of the relative activation barriers.

Path 1 exhibits a transition state at a normalized reaction coordinate of 0.266. The forward activation barrier is 0.422 eV, while the reverse barrier from the subsurface product is 0.953 eV. The reaction energy is −0.531 eV, indicating that the calculated subsurface configuration is thermodynamically more stable than the initial surface adsorption state. The relatively low forward barrier indicates that this surface-to-subsurface migration pathway is kinetically more accessible than the other pathways examined in this study.

In the case of Path 2, the maximum energy point occurs at a normalized reaction coordinate of 0.426. The forward activation barrier is 3.536 eV, while the reverse barrier is 4.426 eV. The reaction energy is −0.891 eV, indicating that the product is thermodynamically more stable than the reactant. However, the substantially larger forward barrier makes this migration route kinetically less accessible than Path 1. The negative reaction energy therefore does not imply that the pathway is kinetically favorable, demonstrating the importance of considering both thermodynamic and kinetic contributions to hydrogen migration.

Path 3 involves a transition state at a normalized reaction coordinate of 0.633. The forward activation barrier is 1.197 eV, while the reverse barrier is 2.140 eV. The reaction energy is −0.942 eV, making the deeper subsurface configuration thermodynamically more stable than the initial surface state. Although the forward barrier is higher than that of Path 1, the negative reaction energy indicates that hydrogen incorporation into the deeper configuration is energetically favorable once the corresponding transition state is overcome.

The relationship between hydrogen adsorption and subsurface migration is particularly relevant for evaluating the hydrogen uptake behavior of the 5B surface. The strongest hydrogen adsorption energy calculated for this surface is −2.793 eV at the B-O bridge site. Despite this strong surface interaction, the calculated Path 1 barrier for migration from the surface to the subsurface is only 0.422 eV, and the resulting subsurface configuration is 0.531 eV lower in energy than the initial surface state. These results indicate that strong hydrogen adsorption on the 5B surface does not inherently prevent subsequent surface-to-subsurface migration along the calculated pathway. The reverse barrier of 0.953 eV is also substantially higher than the forward barrier, indicating a kinetic preference for the calculated subsurface state relative to the initial surface state along this pathway. Thus, the present calculations provide evidence that the strong hydrogen adsorption identified on the 5B surface is compatible with subsurface hydrogen incorporation rather than necessarily resulting in kinetic trapping at the surface.

This result is relevant to the boron concentration selected in the present study. The oxygen-vacancy calculations identified 5B as providing favorable oxygen-removal energetics, and the LST/QST calculations show that this activation advantage is not accompanied by an unfavorable hydrogen migration behavior. The 5B surface therefore retains a kinetically accessible surface-to-subsurface migration pathway, and the calculated adsorption and migration properties do not reveal an inherent incompatibility between strong surface adsorption and subsequent subsurface incorporation.

For comparison, the calculated forward barrier was evaluated against previously reported hydrogen migration barriers in Ti- and V-containing materials. Billeter et al. reported an averaged surface diffusion barrier of 0.5 eV and a bulk diffusion barrier of 0.75 eV for hydrogen in Ti-H, together with an experimental bulk diffusion barrier of 0.53 eV for TiH_≈1.7_ [45]. For ω-TiZr, surface and bulk diffusion barriers of 0.145 eV and 0.386 eV, respectively, have been reported [46]. In disordered Ti-V-Cr alloys, a zero-point-energy-corrected barrier of 0.158 eV was reported for the most probable diffusion pathway [47]. Although these values correspond to different compositions, structural environments, and diffusion pathways, the calculated 0.422 eV barrier is comparable to previously reported hydrogen migration barriers for Ti- and V-containing materials. The comparison therefore provides contextual support for the plausibility of hydrogen migration in the present Ti-V-Nb system.

Collectively, the LST/QST results show that the 5B surface possesses at least one kinetically accessible pathway for hydrogen migration into a thermodynamically more stable subsurface configuration. The results do not establish long-range bulk diffusion or continuous hydrogen storage, which would require additional calculations involving successive subsurface layers and longer-range diffusion pathways. Nevertheless, they demonstrate that the strong hydrogen adsorption predicted for the 5B surface does not preclude surface-to-subsurface hydrogen transport along the calculated pathway. This provides complementary evidence that the 5B composition identified from the oxygen-vacancy analysis can support both activation-related processes and subsequent hydrogen adsorption beyond the surface.

## 4. Conclusions

In this work, the influence of boron surface contamination on the activation behaviour and subsequent hydrogen adsorption on oxidized Ti-V-Nb surfaces was investigated using density functional theory. Systematic screening of boron concentrations from 0 to 66.7 at.% revealed a non-monotonic dependence of oxygen vacancy formation energy on boron content. The 5B configuration (41.7 at.%) exhibited the lowest average oxygen vacancy formation energy of 3.960 eV, representing a reduction of 1.232 eV relative to the pristine oxygen-chemisorbed surface. This result identifies 5B as the least detrimental to activation among the investigated compositions. At 8B (66.7 at.%), the average vacancy formation energy increased to 4.908 eV, indicating partial stabilization of oxygen at high boron loading. Local coordination analysis showed that this non-monotonic trend arises from two competing effects: direct B-O bonding, which weakens adjacent metal–oxygen bonds, and the intrinsic strength of the metal–oxygen bonds, with Ti/V-rich sites easier to activate than Nb-rich sites.

Mulliken analysis revealed distinct behaviour at the two representative sites. At the hollow site, boron became more negative (−0.57 to −0.71 *e*) and Nb more positive (0.30 to 0.66 *e*), whereas at the bridge site boron and oxygen charges remained essentially unchanged (−0.60 to −0.59 *e* and −0.64 to −0.63 *e*), with H instead bonding to nearby Ti, V and Nb atoms at populations of 0.42, 0.41, 0.28 and 0.26. This indicates that boron influences adsorption indirectly by modifying the neighbouring metal centres. EDD maps are consistent with this, showing charge accumulation around H and blue depletion around boron, alongside depletion around Nb at the hollow site, in line with H-H bond cleavage.

PDOS analysis showed B 2*p*–H 1*s* hybridization at the hollow site, while at the bridge site B 2*p* hybridizes with metal *d* states and hydrogen bonds to Ti, V, and Nb. Boron therefore contributes to hydrogen adsorption either through direct B-H bonding or indirectly by modifying the adjacent atoms. The d-band centre shifted progressively downward with increasing boron concentration, from −2.284 eV for the pristine surface to −2.789 eV for the 8B configuration. This trend is consistent with modification of the electronic structure and metal–oxygen interactions by boron contamination. However, the non-monotonic oxygen vacancy formation energies demonstrate that the d-band centre alone cannot describe oxygen removal in the present oxygen-chemisorbed amorphous surface models. Local coordination, boron distribution, and structural reconstruction also play important roles, particularly at high boron concentrations.

The work function increased from 3.126 eV for the pristine surface to 3.344 eV for the 5B configuration, indicating substantial modification of the surface electronic structure following boron contamination. However, the adsorption calculations demonstrate that this electronic modification does not eliminate hydrogen adsorption on the 5B surface. Dissociative H_2_ adsorption remains strongly favourable at bridge and hollow sites. Following H_2_ adsorption, the work function decreased for the investigated adsorption configurations relative to the clean 5B surface. This change indicates a modification of the surface electronic structure upon hydrogen adsorption, although the present calculations do not by themselves establish the magnitude or specific origin of the associated surface dipole change.

The LST/QST calculations further showed that strong hydrogen adsorption on the 5B surface does not inherently prevent subsequent migration into subsurface configurations. The lowest calculated surface-to-subsurface pathway exhibited a forward barrier of 0.422 eV and a reaction energy of −0.531 eV, indicating that the corresponding subsurface configuration is thermodynamically more stable than the initial surface state. The calculated barrier is comparable to previously reported hydrogen migration barriers for Ti- and V-containing materials. These results provide evidence that the strong hydrogen adsorption identified on the 5B surface is compatible with surface-to-subsurface hydrogen migration along the calculated pathway. The calculations do not, however, establish long-range bulk diffusion or continuous hydrogen storage.

Taken together, the results provide an atomistic description linking boron contamination, oxygen removal, hydrogen adsorption, and surface-to-subsurface hydrogen migration on Ti-V-Nb. Among the investigated compositions, 5B provides the lowest average oxygen vacancy formation energy and retains strongly favourable dissociative hydrogen adsorption at specific bridge and hollow sites. Boron contamination modifies the electronic structure but does not eliminate hydrogen adsorption on the selected surface. Hydrogen adsorption was evaluated only on a single concentration selected from the oxygen-vacancy screening, so an independent hydrogen-adsorption optimum across boron concentrations cannot be established from the present data. Future calculations at other concentrations would be needed to determine whether this composition remains the least detrimental across all performance criteria. The calculated trends are consistent with experimental observations from the Large Helical Device, where boronization did not impair NEG pumping performance [18]. Experimental studies are required to validate the predicted concentration dependence under reactor-relevant conditions.

## Figures and Tables

**Table 1:** Boron concentration series and corresponding physical interpretation. The concentration is defined as the number of added boron atoms divided by the 12 metal atoms in the topmost surface layer of the amorphous slab.

$N_{B}$	Physical Meaning	Boron Concentration (at.%)
0	clean surface	0.0
1	dilute perturbation regime	8.3
3	moderate alloying regime	25.0
5	strong alloying regime	41.7
8	very strong surface reconstruction regime	66.7

**Table 2:** Comparison of oxygen vacancy formation energies for oxidized Ti-V-Nb(100) and boron-contaminated oxidized Ti-V-Nb(100) surfaces at different boron concentrations.

Vacancy Site	0 B (0.0 at.%)	1 B (8.3 at.%)		3 B (25.0 at.%)		5 B (41.7 at.%)		8 B (66.7 at.%)	
	$E_{\mathrm{vac}}$ (eV)	$E_{\mathrm{vac}}$ (eV)	ΔE_vac_ (eV)	$E_{\mathrm{vac}}$ (eV)	ΔE_vac_ (eV)	$E_{\mathrm{vac}}$ (eV)	ΔE_vac_ (eV)	$E_{\mathrm{vac}}$ (eV)	ΔE_vac_ (eV)
O1	5.802	5.774	−0.027	5.980	+0.178	5.611	−0.191	4.986	−0.816
O2	5.156	5.107	−0.050	5.209	+0.053	3.155	−2.001	5.461	+0.305
O3	5.593	5.610	+0.016	5.359	−0.234	5.221	−0.372	5.285	−0.308
O4	4.751	3.769	−0.983	4.424	−0.327	3.015	−1.736	4.348	−0.403
O5	5.195	4.116	−1.079	5.302	+0.107	3.537	−1.658	4.857	−0.338
O6	5.164	5.145	−0.019	5.328	+0.164	5.047	−0.117	5.060	−0.104
O7	4.597	5.179	+0.582	3.705	−0.892	3.018	−1.579	4.462	−0.135
O8	5.282	5.153	−0.129	4.672	−0.610	3.078	−2.204	4.805	−0.477
Minimum	4.597	3.769	−1.079	3.705	−0.892	3.015	−2.204	4.348	−0.816
Maximum	5.802	5.774	+0.582	5.980	+0.178	5.611	−0.117	5.461	+0.305
Average	5.193	4.982	−0.211	4.997	−0.196	3.960	−1.232	4.908	−0.285

**Table 3:** Local coordination of oxygen sites at 3B, 5B, and 8B, with the number of B and Nb neighbors.

Site	3B			5B			8B		
	Coordination	#B	#Nb	Coordination	#B	#Nb	Coordination	#B	#Nb
O1	Ti-V-Nb-Nb-Nb	0	3	Ti-V-Nb-B	1	1	Ti-V-Nb-Nb-Nb-B	1	3
O2	Ti-V-V-Nb-Nb	0	2	Ti-V-V-Nb	0	1	Ti-V-V-Nb-Nb	0	2
O3	Ti-Ti-V-Nb-Nb	0	2	Ti-Ti-V-Nb-Nb-B	1	2	Ti-Ti-V-Nb-Nb	0	2
O4	Ti-V-V-V	0	0	Ti-V-V-V	0	0	Ti-V-B-B	2	0
O5	Ti-V-V-V-V	0	0	Ti-V-V-V-V	0	0	Ti-V-V-V-V	0	0
O6	V-V-V-Nb-Nb	0	2	V-V-V-Nb-Nb	0	2	Ti-Ti-V-V-V-B	1	0
O7	V-Nb-Nb-B-B	2	2	V-Nb-Nb-B-B-B	3	2	V-Nb-Nb-B-B	2	2
O8	V-V-V-Nb-Nb-B	1	2	V-V-Nb-Nb-B	1	2	V-V-V-Nb-Nb-Nb-B	1	3

**Table 4:** Hydrogen adsorption properties on the 5B boron-contaminated oxidized Ti-V-Nb surface selected for activation.

Top Sites			Bridge Sites			Hollow Sites		
Site	d_H−H_	$E_{\mathrm{ads}}$	Site	d_H−H_	$E_{\mathrm{ads}}$	Site	d_H−H_	$E_{\mathrm{ads}}$
T1	0.759	−0.049	B1	2.162	−2.224	H1	3.936	−2.595
T2	0.758	−0.043	B2	2.588	−1.765	H2	2.574	−2.237
T3	0.839	−0.484	B3	2.789	−1.765	H3	2.064	−2.543
T4	0.759	−0.035	B4	2.403	−1.815	H4	1.989	−0.972
T5	0.757	−0.072	B5	3.897	−1.393	H5	2.310	−1.633
T6	0.757	−0.059	B6	3.554	−2.793	H6	3.357	−1.361
			B7	2.116	−2.697	H7	4.018	−1.380
			B8	2.692	−2.270	H8	2.305	−2.657
			B9	3.626	−2.648			

**Table 5:** Mulliken population analysis for H_2_ adsorption on the 5B boron-contaminated oxidized Ti-V-Nb surface before and after adsorption.

Adsorption Site	Atom	Charge (*e*)		Bonds	Bond Population	*d* (Å)
		Before	After			
T3 (Nb)	Nb	0.33	0.38	Nb-H1	−0.15	1.988
				Nb-H2	−0.09	2.018
	H1	–	−0.12	H1-H2	0.97	0.839
	H2	–	−0.14			
B7 (direct) (B-V)	B	−0.57	−0.65	B-H1	0.55	1.356
				B-H2	1.11	1.217
	V	0.61	0.85	V-H1	−0.08	1.806
				V-H2	0.01	2.536
	H1	–	−0.28	H1-H2	−0.07	2.117
	H2	–	−0.05			
B6 (indirect) (B-O)	B	−0.60	−0.59	B-H1	0.04	2.080
				B-H2	−0.04	2.401
	O	−0.64	−0.63	O-H1	−0.01	2.834
				O-H2	0.00	2.942
Adjacent metals	Ti	0.63	0.64	Ti-H2	0.42	1.949
	V	0.76	0.72	V-H2	0.41	1.827
	V	−0.18	−0.07	V-H1	0.28	2.010
	Nb	0.21	−0.04	Nb-H1	0.26	1.925
	Ti	0.45	0.44	Ti-H1	0.11	1.948
	H1	–	−0.25	H1-H2	–	3.554
	H2	–	−0.28			
H8 (Nb-B-B)	Nb	0.30	0.66	Nb-H1	0.06	1.925
				Nb-H2	−0.15	2.124
	1B	−0.57	−0.71	1B-H1	0.05	2.251
				1B-H2	1.00	1.257
	2B	−0.32	−0.17	2B-H1	0.23	1.713
				2B-H2	0.05	2.537
	H1	–	−0.19	H1-H2	−0.03	2.305
	H2	–	−0.10			

## Data Availability

Data available on request from the corresponding author due to institutional restrictions and ongoing related research.
